# The Impact of Retail-Sector Delivery of Artemether–Lumefantrine on Malaria Treatment of Children under Five in Kenya: A Cluster Randomized Controlled Trial

**DOI:** 10.1371/journal.pmed.1000437

**Published:** 2011-05-31

**Authors:** Beth P. Kangwana, Sarah V. Kedenge, Abdisalan M. Noor, Victor A. Alegana, Andrew J. Nyandigisi, Jayesh Pandit, Greg W. Fegan, James E. Todd, Simon Brooker, Robert W. Snow, Catherine A. Goodman

**Affiliations:** 1 Malaria Public Health & Epidemiology Group, Kenya Medical Research Institute - Wellcome Trust Research Programme, Kenya; 2 Centre for Tropical Medicine, Nuffield Department of Clinical Medicine, University of Oxford, Oxford, United Kingdom; 3 Division of Malaria Control, Ministry of Public Health and Sanitation, Nairobi, Kenya; 4 Pharmacy and Poisons Board, Nairobi, Kenya; 5 London School of Hygiene & Tropical Medicine, London, United Kingdom; University of Melbourne, Australia

## Abstract

**Background:**

It has been proposed that artemisinin-based combination therapy (ACT) be subsidised in the private sector in order to improve affordability and access. This study in western Kenya aimed to evaluate the impact of providing subsidized artemether–lumefantrine (AL) through retail providers on the coverage of prompt, effective antimalarial treatment for febrile children aged 3–59 months.

**Methods and Findings:**

We used a cluster-randomized, controlled design with nine control and nine intervention sublocations, equally distributed across three districts in western Kenya. Cross-sectional household surveys were conducted before and after the delivery of the intervention. The intervention comprised provision of subsidized packs of paediatric ACT to retail outlets, training of retail outlet staff, and community awareness activities. The primary outcome was defined as the proportion of children aged 3–59 months reporting fever in the past 2 weeks who started treatment with AL on the same day or following day of fever onset. Data were collected using structured questionnaires and analyzed based on cluster-level summaries, comparing control to intervention arms, while adjusting for other covariates. Data were collected on 2,749 children in the target age group at baseline and 2,662 at follow-up. 29% of children experienced fever within 2 weeks before the interview. At follow-up, the percentage of children receiving AL on the day of fever or the following day had risen by 14.6% points in the control arm (from 5.3% [standard deviation (SD): 3.2%] to 19.9% [SD: 10.0%]) and 40.2% points in the intervention arm (from 4.7% [SD: 3.4%] to 44.9% [SD: 11.7%]). The percentage of children receiving AL was significantly greater in the intervention arm at follow-up, with a difference between the arms of 25.0% points (95% confidence interval [CI]: 14.1%, 35.9%; unadjusted *p* = 0.0002, adjusted *p* = 0.0001). No significant differences were observed between arms in the proportion of caregivers who sought treatment for their child's fever by source, or in the child's adherence to AL.

**Conclusions:**

Subsidizing ACT in the retail sector can significantly increase ACT coverage for reported fevers in rural areas. Further research is needed on the impact and cost-effectiveness of such subsidy programmes at a national scale.

**Trial Registration:**

Current Controlled Trials ISRCTN59275137 and Kenya Pharmacy and Poisons Board Ethical Committee for Clinical Trials PPB/ECCT/08/07.

*Please see later in the article for the Editors' Summary*

## Abstract 2

In a cluster randomized trial, Beth Kangwana and colleagues find that provision of subsidized packs of the malaria therapy artemether-lumefantrine to shops more than doubled the proportion of children with fever who received drugs promptly.

## Editors' Summary

**Background:**

Malaria is a major global public-health problem. Half the world's population is at risk of this mosquito-borne parasitic disease, which kills a million people (mainly children living in sub-Saharan Africa) every year. Although several parasites cause malaria, *Plasmodium falciparum* is responsible for most of these deaths. For the past 50 years, the main treatments for malaria have been drugs such as sulfadoxine–pyrimethamine and chloroquine. Unfortunately, parasitic resistance to these inexpensive "monotherapies" is now widespread and there has been an upsurge in the illness and death caused by *P. falciparum*. To combat this increase, the World Health Organization (WHO) now recommends artemisinin-based combination therapy (ACT) for first-line treatment of *P. falciparum* malaria in all regions with drug-resistant malaria. In ACT, artemisinin derivatives (new, fast-acting antimalarial drugs) are used in combination with another antimalarial to reduce the chances of *P. falciparum* becoming resistant to either drug.

**Why Was This Study Done?:**

Despite WHO's recommendation, ACT use in many developing countries remains low partly because of its high retail price. To increase the affordability of and access to ACT, the Global Fund to Fight AIDS, Tuberculosis and Malaria is planning to run an ACT subsidy mechanism called the “Affordable Medicines Facility – malaria” (AMF-m). Using money provided by various donors, the Global Fund aims to reduce the private sector retail costs of ACT to those of monotherapies by making "copayments" directly to ACT manufacturers. Phase I of the AMF-m is already being implemented in pilots in several countries, but there are few data on the likely impact of private sector ACT subsidies on the coverage of prompt, effective treatment at the community level. In this cluster randomized controlled trial, the researchers investigate the impact of an intervention package that includes ACT subsidies on malaria treatment of young children in a high malaria transmission area of western Kenya. In a cluster randomized controlled trial, groups of patients rather than individual patients are randomly assigned to receive a test or control intervention, and the outcomes in different clusters are compared.

**What Did the Researchers Do and Find?:**

The researchers randomly assigned 18 rural sublocations (the lowest administrative level in Kenya) to receive the intervention—the provision of subsidized packs of the ACT artemether-lumefantrine (AL) to retail outlets, retail staff training, and community awareness activities—or to act as controls. The researchers collected data about recent fever (a symptom of malaria) in children aged 3–59 months and its treatment with AL from randomly selected households in the intervention and control sublocations 4 months before and 8 months after roll-out of the intervention. At follow-up, 19.9% of children in the control arm received AL within 24 hours of fever developing compared to 5.3% of children at baseline (a 14.5% point rise). In the intervention arm, the percentage of children receiving AL within 24 hours of fever developing increased from 4.7% at baseline to 44.9% at follow-up (a 40.2% point rise). Moreover, the proportion of children receiving AL in the intervention arm was significantly greater than in the control arm (that is, unlikely to have happened by chance). Put another way, the intervention more than doubled the proportion of children with fever who received AL promptly.

**What Do These Findings Mean?:**

These findings show that in the rural areas of Kenya included in this study, the provision of subsidized ACT in the private retail sector can significantly increase the coverage of prompt and effective treatment of fever in children with ACT; the increase in ACT coverage in the control arm probably reflects improved availability of AL in public-health facilities. However, these findings may not be generalizable to other settings and, because the design of this trial and that of the planned AMF-m roll-out are somewhat different (through AMF-m, subsidized drugs will be available to all age groups, for example), these results must be used with caution when trying to predict the outcome of AMF-m. Most importantly, the tested intervention only achieved prompt ACT uptake in 44.9% of children with fever, somewhat lower than the target of 80% set by the Roll Back Malaria Partnership. Thus, although the provision of subsidized ACTs is likely to improve ACT coverage, additional strategies to increase the prompt use of ACT need to be identified.

**Additional Information:**

Please access these Web sites via the online version of this summary at http://dx.doi.org/10.1371/journal.pmed.1000437.

- Information is available from the World Health Organization on malaria (in several languages); the 2010 World Malaria Report provides details of the current global malaria situation
- The US Centers for Disease Control and Prevention provide information on malaria (in English and Spanish)
- Information is available from the Roll Back Malaria Partnership on the global control of malaria including fact sheets about ACT and about malaria in Kenya, and information on AMF-m
- The Global Fund to Fight AIDS, Tuberculosis and Malaria, an international financing institution that invests the world's money to save lives, also has information on fighting malaria and on the AMF-m (in several languages)
- MedlinePlus provides links to additional information on malaria (in English and Spanish)

## Introduction

Artemisinin-based combination treatments (ACTs) are generally accepted as the best treatment for uncomplicated *Plasmodium falciparum* malaria, as they have been shown to be highly effective and generally well tolerated [1]. Consequently all *P. falciparum*–endemic countries in Africa have adopted ACTs as national policy, but usage remains very low, with only 16% of febrile children under the age of 5 years receiving ACTs in 2008 [2]. A large gap therefore exists between the target, set by the Roll Back Malaria Partnership, that 80% of malaria cases be treated with effective treatment within 24 hours, and the situation on the ground [2].

There have been calls for radical solutions to improve access to effective malaria treatment. Prominent among these is a proposal to subsidize ACTs in the private sector [3]. The private sector is an important source of malaria drugs [4],[5], but the high retail price of ACTs has resulted in continued use of more affordable but less effective antimalarial drugs in this sector such as sulphadoxine–pyrimethamine (SP) and amodiaquine [6].

An ACT subsidy mechanism known as the Affordable Medicines Facility-malaria (AMF-m) is currently being established, managed by the Global Fund for HIV/AIDS, TB and Malaria. The Global Fund will make copayments directly to preselected ACT manufacturers, lowering the import cost for both public and private sector buyers. The aim is to reduce ACT retail prices to a level similar to less effective antimalarials, to increase demand for ACTs and displace monotherapies and substandard treatments from the market. Additional funding is to be made available to countries for “supporting interventions” such as community awareness, provider training, and regulatory strengthening. AMF-m is scheduled to roll out in eight Phase One countries (Cambodia, Ghana, Kenya, Madagascar, Niger, Nigeria, Uganda and the United Republic of Tanzania [mainland and Zanzibar]) [7].

Limited experience with private-sector ACT subsidies indicates that they can lead to increased ACT uptake and decreased monotherapy use [8],[9]. No data, however, are available on the impact on the key outcome of coverage of prompt effective treatment of fever at the community level. With only a subset of the community using retail outlets, it is not clear if an intervention targeting retailers only will demonstrate a significant effect on overall treatment coverage. In addition, there are concerns that shopkeepers may not stock the subsidized medicines due to capital constraints; that brief training may be insufficient to change treatment practices; and that retailers may not pass on the subsidy to the consumer, preferring instead to maximize their profits. Also it is not known whether caretakers of young children will be willing to change their treatment practices and to trust shopkeepers to provide good-quality ACTs. Finally, there are concerns that the subsidies will be taken advantage of by the relatively well-off, with the poorest in the community unable to afford even the subsidized ACTs [9]–[14].

Here we report a cluster randomised trial to address these gaps in knowledge, evaluating the impact of a package including ACT subsidies, retailer training, and community awareness on ACT coverage, price, and adherence in a high malaria transmission area of western Kenya.

## Methods

### Ethical Approval

Ethical approval was obtained from the Kenya Medical Research Institute (KEMRI) Ethical Review Committee (# 1361), the Kenya Pharmacy and Poisons Board Ethical Committee for Clinical Trials (# PPB/ECCT/08/07), and the London School of Hygiene and Tropical Medicine Ethical Review Committee (# 5288). The study is registered with Current Controlled Trials (# ISRCTN59275137). Written consent was obtained from the household heads or a representative, and verbal consent was obtained from all caregivers interviewed. Ethics statement: “We (the KEMRI National Ethics Review Committee) acknowledge the receipt of Teso, Samia and Wanga- translated Informed Consent Documents. The committee is satisfied with the contents which assures the understanding of potential research participants. The study is hereby granted [ethical] approval.”

### Study Overview

The study was conducted in Kenya, where the first-line antimalarial for uncomplicated cases is artemether–lumefantrine (AL). AL is a prescription-only medicine, officially available at registered health facilities and pharmacies only, although in practice many prescription-only drugs are dispensed without a prescription in pharmacies and other retail outlets. It has a private sector retail price of around 6.16 US dollars (USD) (500 Kenya Shillings [KSH]), compared with an average of around 0.37 USD for common older antimalarials such as SP and amodiaquine (based on USD-to-KSH exchange rate for 1st November 2008 when the subsidized drugs were first distributed [15]). As a comparison, in Kenya, the poverty line (the cost of a basic basket of food and non-food items) in 2003 was about 1,239 KSH (15.25 USD) per person per month for rural inhabitants [16]. The pilot was implemented by a team from Division of Malaria Control in the Kenyan Ministry of Public Health and Sanitation, Population Services International (PSI), and the Pharmacy and Poisons Board (PPB).

### Study Sites

The study was conducted in three districts in Kenya's Western Province: Busia, Butere-Mumias, and Teso (for maps of study areas see Figures S1 and S2). These areas were selected because of their high malaria endemicity [17], the presence of a relatively active retail market, and the absence of other retail sector malaria treatment interventions.

At the time of the survey, the percentage of the population living below the poverty line in the study districts averaged 67% in Busia, 62% in Butere-Mumias, and 50% in Teso. Population densities per km^2^ were 433, 611, and 406 in Busia, Butere-Mumias, and Teso, respectively [18]. This area suffers from the highest malaria prevalence in Kenya, with *Plasmodium falciparum* parasitaemia prevalance in children aged 2–10 years being 40% or more [17]. At the time of the survey, Butere-Mumias had 51 government health facilities, Busia 39, and Teso 21, consisting of dispensaries, health centres, and one district hospital per district [19]. All government health facilities in Kenya are supposed to supply AL free to patients, although stock-outs and unofficial fees are common [20],[21]. Malaria diagnosis is predominantly presumptive, based on the presence of fever, in both public and private health sectors [22],[23].

### Study Design

We employed a cluster randomised controlled design, collecting data before (at baseline) and after (at follow-up) the roll out of the intervention. Randomization was conducted at the sublocation level, which is the fifth and lowest administrative level in Kenya, governed by a subchief. To be included in the sampling frame the sublocations had to have populations between 2,500 and 10,000; smaller sublocations were excluded to ensure there was a reasonable scale for implementation and adequate sample sizes for the evaluation; larger sublocations were excluded to contain the costs. Urban and periurban sublocations, which represented between a quarter and a third of all sublocations in the study districts, were excluded from the sampling frame because of the high likelihood of contamination when people from surrounding sublocations travelled to purchase antimalarials in urban areas. A modified randomization process was used to select sublocations. A random list of all eligible sublocations was formulated per district in Microsoft Excel. The first intervention sublocation was selected from the top of the list. In order to reduce the potential for contamination a “buffer zone” was created where all sublocations located within two sublocation boundaries of the selected sublocation were removed from the list. The list was reshuffled randomly and the first sublocation on the new list allocated to the control arm. The same procedure of creating a buffer zone around this sublocation was carried out, and the list again randomly reshuffled and a second intervention sublocation selected. This process was continued, alternating between the selection of intervention and control sublocations, until three intervention and three control sublocations had been selected within the district. The estimated population in the control and intervention arms were 38,620 and 44,538, respectively (average population per selected sublocation of 4,620, range 2,703 to 9,294) [18]. Due to the public information campaign around the subsidised drugs in the intervention arm, blinding was not possible for shopkeepers, community members, or data collectors.

### The Intervention

The three main components of the intervention were provision of subsidized packs of paediatric ACT to retail outlets, training of retail outlet staff, and community awareness activities. No interventions were implemented in the control arm. In both intervention and control arms the policy of provision of free AL at government facilities continued unchanged. In 2006/7 the government had carried out AL awareness campaigns across the country, so both arms had previously received some general information on the current malaria treatment policy (personal communication, Andrew Nyandigisi, Division of Malaria Control, Ministry of Public Health and Sanitation Kenya).

The intervention targeted retail outlets serving intervention arms, which were identified through an outlet census. Outlets were included in the census if they were located in or on the borders of the intervention sublocations and identified by key informants as serving their populations. An initial list of retail outlets was sourced from local public health officers, and updated with input from local chiefs and subchiefs. The list was further amended after walking around the study areas with village elders to confirm the presence of outlets and to add missed outlets. The snowball technique [24] was then used where each shop visited was asked about the presence of other outlets in their area. Finally, members of the community passing by were opportunistically asked about the location of outlets.

**Figure 1:**
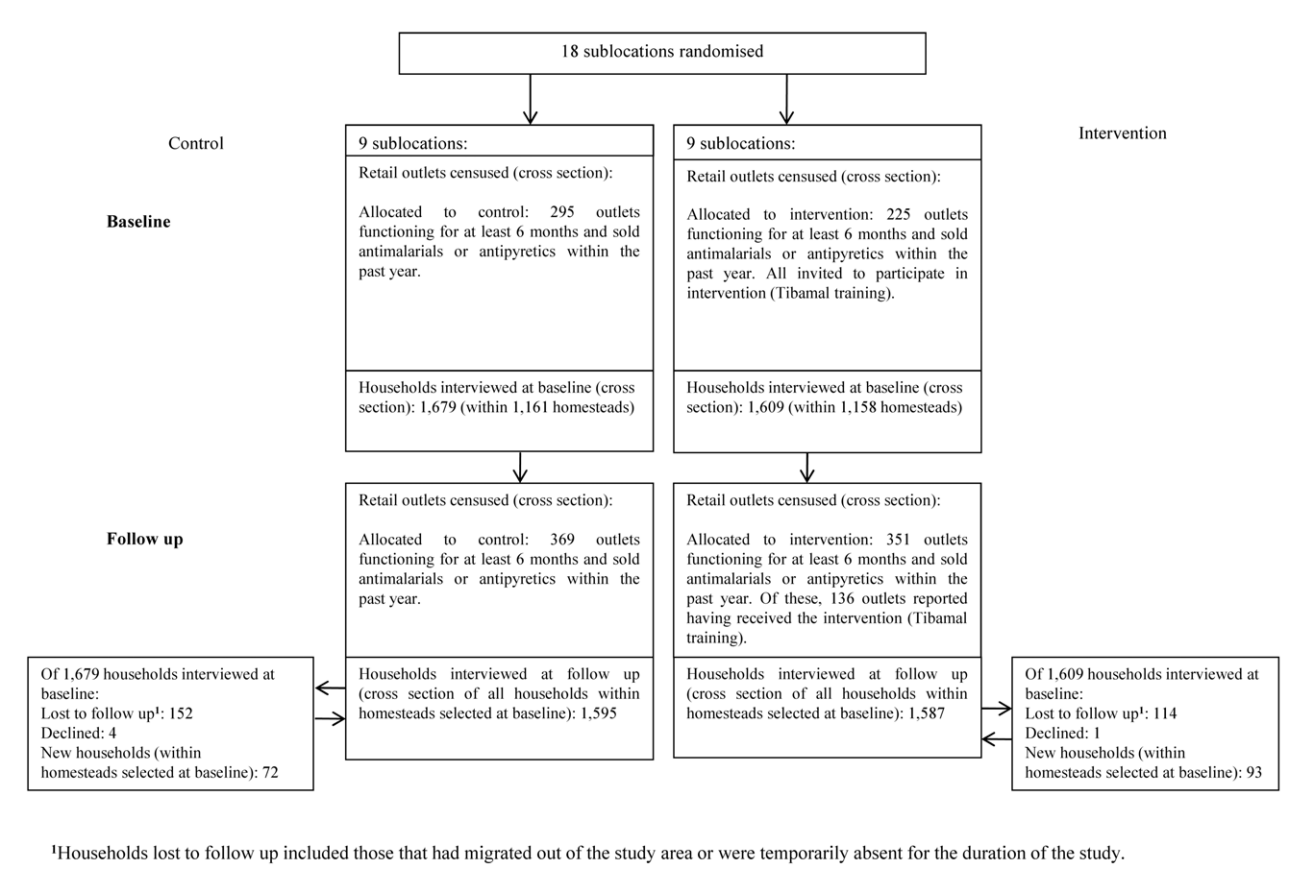
Flow diagram showing households and retail outlets sampled and interviewed.

Enumerated outlets were invited for training if they had been functioning for a minimum period of six months and were selling antimalarials and/or antipyretics during the past year. A total of 225 outlets were deemed eligible for training, of which 61 were specialised drug stores (registered or unregistered pharmacies) and 164 general stores (which sold medicines alongside general household goods). Outlet staff attended a one-day malaria-related training between August and October 2008 covering clinical diagnosis, treatment, adverse drug reactions (ADRs), and patient referral. Training materials were developed by the implementation team, building on those used previously for shopkeeper training in Kenya [25]. At follow-up in the intervention arm, 320 outlets met the above eligibility requirements and were successfully interviewed, of which 136 reported having at least one staff member trained (43%) (Figure 1) [26].

From November 2008, subsidised AL was provided to trained retail outlets in packs of six tablets (for children aged 3–35 months) and 12 tablets (for children aged 36–59 months). The AL was branded as Tibamal, a pretested name derived from the Kiswahili words “Tiba ya Malaria,” meaning *malaria cure,* and came with patient instructions suitable for those with low literacy levels. Kiswahili is one of the official languages of Kenya which is commonly understood by all tribal groups in the country, including those participating in the study. The PPB granted special dispensation for AL to be dispensed over the counter in the intervention arm. PSI sales staff delivered the treatment directly to the trained outlets on a monthly basis, and shopkeepers purchased the treatment at a wholesaler price of 0.10 USD (8 KSH) per pack (both packs were the same price). The outlets were instructed to sell the packs at a retail price of 0.25 USD (20 KSH), and this price was printed on the drug packaging. This provided a retail mark-up of 0.15 USD (12 KSH) per pack. The intervention was designed to give Tibamal a 150% retailer markup, exceeding that of other popular antimalarials such as amodiaquine and SP, which generally have mark-ups of 50%–100%.

At baseline AL was stocked in only 0.5% of outlets in the control arm and 2.4% in the intervention arm. At follow-up, AL (including Tibamal) was stocked by 37.6% of outlets in the intervention arm but only 5.5% in the control arm. No stocks of Tibamal were found in the control arm; however, in the intervention arm, Tibamal was present in 35% of outlets. In the subsample of Tibamal trained outlets, 72% were found to be stocking AL, 69% of which was Tibamal branded AL. The median cost of a tablet of AL at baseline was 0.18 USD in the control arm; by follow-up this had fallen slightly to 0.14 USD. In the intervention arm the cost of an AL tablet fell from 0.15 USD at baseline to 0.04 USD at follow-up, a difference of 0.11 USD. Availability of other ACTs in retail outlets was rare at both time points. The price for other ACTs was similar to commercial sector AL [26].

Trained outlets were supplied with job aids, consisting of a referral flow chart and dosing guidelines, to support dispensing. Shopkeepers were also supplied with a Daily Activity Register to document AL dispensed, and forms for referring severe cases and suspected ADRs to local health facilities. Copies of completed referral forms were to be collected by PSI sales staff and forwarded to the PPB. All supporting materials supplied to outlets were provided free of charge. A follow-up supervisory visit was made by the implementation team 3 months after the initial supplies to monitor outlet practices.

The main community awareness activities began in March 2009, and then intermittently in August and September 2009. Activities were to continue to the end of the pilot in May 2010. They consisted of nine community leader workshops that targeted 47 people; nine community events carried out by PSI that targeted 11,500 people; ten small group discussions that targeted 200 people; and outreaches carried out by community-based organisations that targeted 21,000. These activities were designed to make the community aware of malaria, the availability of Tibamal, and the importance of adherence to the medication. Tibamal was also advertised through posters and paintings on shops that sold the treatment. Tibamal branded headscarves, t-shirts, and pens were also freely distributed to the intervention community.

### Data Collection

The primary outcome was defined as the proportion of children aged 3–59 months reporting fever in the past 2 weeks who started treatment with AL on the same day or following day of fever onset. Secondary outcomes included the adequacy of AL doses obtained and consumed, and the price paid per pack. These were assessed using pre- and post-household surveys conducted in July–August 2008 and July–August 2009. The study was based on an intention to treat analysis where clusters were not adjusted or further selected depending on the proportion of retail outlets which actually received the intervention. The sample size was based on detecting a 20% point difference in the primary outcome, with 5% significance, 80% power, and an estimated design effect of 2 to account for the cluster survey design (percentage point refers to the absolute difference observed between two percentages, in this case between the outcome percentages observed between the intervention and control arm). We estimated that the primary outcome would be 20% at baseline (based on data collected by Gitonga et al. [27], and allowing for some increase since that survey took place). A design effect of 2 was considered conservative based on an intra-class correlation coefficient of 0.16 from a similar previous survey in Kenya [28], and an estimated 43 homesteads per cluster. This led to a required sample size of 158 childhood fevers in each arm, which we estimated would require data collection from 1,138 homesteads in each arm, equivalent to around 210 households per sublocation. A homestead is a group of households within the same compound belonging to a single extended family. A household consists of a person or a group of related or unrelated persons who live together in the same dwelling unit, who acknowledge one male or female as the head of the household, who share the same housekeeping arrangements, and who are considered to constitute one unit. A homestead can contain one or more households.

Three enumeration areas (EAs) were randomly selected within each intervention and control sublocation on the basis of probability proportional to population size. A homestead census was carried out in the selected EAs in May 2008 and each homestead was mapped using GPS hand-held receivers (Garmin etrex and Trimble 12 band GPS units). From the homesteads enumerated, an average of 43 were randomly selected using simple randomisation with Excel 2007, within each EA. To achieve the sample size, homesteads selected for sampling but not available during data collection were replaced by the next available from a randomly ordered list of homesteads, formulated during the census. A pretested questionnaire was administered to all household heads within the selected homesteads to ascertain household socioeconomic status, and to all caregivers of children under 5 years of age reporting fever episodes in the 2 weeks prior to the interview to assess treatment-seeking behaviour and medicine use. All homesteads agreeing to participate at baseline were revisited at follow-up. All households within each homestead were interviewed at each time point, including new households that were established at follow-up.

### Data Analysis

Baseline data were captured on paper questionnaires and double entered into Microsoft Access (2007). Follow-up data were captured using personal digital assistants and Pendragon Forms version 5.1 (Pendragon Software Corporation, Libertyville, Illinois [http://www.pendragon-software.com] and downloaded onto Microsoft Access [2007]). The data were analyzed in STATA (College Station, Texas) by a two-stage process, with baseline and postintervention data analyzed separately. In the first stage a summary cluster measure was obtained for each cluster. The second stage involved comparing the sets of cluster-specific measures in control and intervention arms at follow-up using the unpaired *t*-test [29]. A crude analysis was carried out on the cluster summaries using the simple two tailed t-test to obtain the means, 95% confidence intervals (CIs) and standard deviations (SDs) for the outcome of interest. In addition, an adjusted analysis was carried out at follow-up on all indicators using an individual level logistic regression run on the pooled data set (control and intervention arms). To control for potential confounders, the following covariates were considered: patient age and sex, caretaker's and household head's education level, wealth score, bed net use last night, district, and, when adjusting for the adequacy of AL doses obtained and consumed, the source of treatment. All covariates significant at a *p*-value of 0.2 in the bivariate analysis were entered into the regression model. Baseline values for the outcome in question were also included as covariates if a difference of 5% points or more was observed between the arms at baseline. The intervention status of the cluster was not included in the logistic regression model. Rather, the regression model provided the predicted outcome in the absence of the intervention effect. Mean predicted and observed outcomes were obtained per cluster and residuals were obtained by subtracting the predicted outcomes from those observed in each cluster. The *t*-test was used on these residuals to assess the intervention effect, adjusted for the covariates included in the logistic regression model. The *t*-test was used for both crude and adjusted analyses, as it has been shown to be highly robust even for small numbers of clusters. A separate analysis allowing for clustering within homesteads was also conducted.

The presence of certain household assets (selected on the basis of those included in the 2003 Kenyan Demographic and Health survey [30]) was recorded to assess the wealth of the household. A wealth index was constructed by assigning weights to each asset using principal components analysis (PCA) with weights based on the first principal component only [31]. Each household was then assigned to a specific wealth quintile, from most poor through to the least poor. All interviewed households were included in the PCA, regardless of whether they contained children aged under 5. The PCA was conducted separately for baseline and follow-up surveys. In the analysis we tested for heterogeneity in the effect of the intervention across the wealth quintiles using ANOVA on the cluster percentages for the primary outcome.

## Results

### Characteristics of Sampled Children

We completed interviews in 2,319 homesteads at baseline (3,288 households), and 2,204 homesteads at follow-up (3,182 households). Data were collected on 2,749 children aged 3–59 months at baseline (1,381 and 1,368 in the control and intervention arms respectively), and 2,662 at follow-up (1,305 and 1,357 respectively) (Table 1). Around half the children were male. Just under half had slept under an insecticide-treated net (ITN) the night before the interview at baseline, and just over half at follow-up. Reported fever within 2 weeks prior to the interview ranged from 26% in the control arm at baseline to 32% in the intervention arm at follow-up. Around half the household heads for the sampled children had completed primary school or above. Within each arm, sampled children were relatively equally distributed across the different wealth quintiles. Fewer homesteads needed to be visited to find one childhood fever than originally estimated, resulting in more fevers being detected than expected from the sample size calculations.

**Table 1:** Characteristics of surveyed children aged 3–59 months (mean of cluster summaries from the nine intervention and nine control clusters).

Characteristic	Baseline		Follow-Up	
	Control, % (SD)	Intervention, % (SD)	Control, % (SD)	Intervention, % (SD)
Total children present in interviewed households	1,381	1,368	1,305	1,357
Percentage of children aged ≥36 months	40.6 (3.8)	39.6 (2.1)	43.1 (4.1)	42.1 (3.3)
Male	50.5 (3.6)	53.1 (3.9)	51.6 (3.4)	52.1 (2.9)
Household heads had completed primary school or above	54.7 (8.5)	47.8 (6.9)	53.2 (9.4)	47.5 (8.4)
Slept under an ITN last night	49.7 (9.2)	46.2 (5.6)	57.1 (7.7)	57.8 (10.3)
Wealth quintile^a^				
Quintile 1 (most poor)	20.6 (8.9)	21.9 (6.3)	20.1(8.6)	23.6 (7.2)
Quintile 2 (very poor)	22.7 (9.3)	21.3 (7.6)	22.3 (8.2)	23.2 (8.8)
Quintile 3 (poor)	18.0 (3.8)	21.0 (4.5)	19.0 (5.0)	20.1 (5.7)
Quintile 4 (less poor)	19.6 (6.8)	19.8 (7.2)	18.7 (10.6)	19.5 (9.7)
Quintile 5 (least poor)	19.1 (6.9)	16.0 (4.5)	19.9 (8.7)	13.3 (4.6)
Fever prevalence within the past 2 weeks	26.0 (8.6)	30.3 (8.7)	27.0 (7.4)	32.4 (10.3)

a Wealth quintiles are based on all households interviewed. The percentages represent the number of households with children 3-59 months that fall within each quintile.

### Treatment-Seeking Behaviour

More than 86% of children who experienced a fever within 2 weeks of the interview had some kind of action taken by the caregiver to treat the fever, with no significant difference seen at follow-up across the two arms (Table 2). A total of 779 actions were taken at baseline across both arms, and 728 at follow-up (some caregivers took more than one action for a given fever). Of all actions taken, the most common were visits to government facilities and specialised drug stores (each accounting for around a third of actions) (Table 3). These were followed by visits to general stores and missionary/private health facilities, with use of traditional healers very rarely reported. At follow-up, there was no significant difference in the kind of actions taken across the two arms. An increase was seen in the number of visits to general stores and a decrease in visits to specialised drug outlets from baseline to follow-up; however, this change in behaviour was observed in both arms. When the analysis was restricted to first actions only, similar patterns were observed.

**Table 2:** Antimalarial treatment obtained for children aged 3-59 months with fever in the previous 2 weeks (a comparison of the nine intervention and nine control clusters).

Treatment-Seeking Behaviour Outcomes	Control^a^ (N = 9), % (SD)	Intervention^b^ (N = 9), % (SD)	Difference in Means (95% CI)	*p*-Value^c^, Unadjusted; *Adjusted*
Children who had care sought for them after developing fever:				
Baseline	86.6 (6.4)	90.1 (4.7)		
Follow-up	88.9 (4.3)	89.1 (4.9)	0.2 (4.8, −4.4)	0.9304; *0.8759*
Children who received an antimalarial:				
Baseline	38.9 (7.8)	45.5 (9.4)		
Follow-up	50.3 (11.8)	64.0 (10.5)	13.7 (2.5, 24.9)	0.0192; *0.0074*
Children who received an antimalarial monotherapy:				
Baseline	29.8 (11.1)	39.0 (7.7)		
Follow-up	22.8 (7.8)	12.4 (4.8)	−10.4 (−3.9, −16.9)	0.0036; *0.0518* d
Children who received any brand of AL:				
Baseline	9.8 (8.3)	7.7 (5.1)		
Follow-up	27.3 (15.2)	53.7 (12.3)	26.4 (12.6, 40.2)	0.0009; *0.0001*
Children who received Tibamal:				
Baseline	0 (0)	0 (0)		
Follow-up	0 (0)	33.7 (6.8)	33.7 (28.8, 38.5)	0.0001; *0.0001*
Children who received any brand of AL on the same day or following day of fever onset:^e^,^f^				
Baseline	5.3 (3.2)	4.7 (3.4)		
Follow-up	19.9 (10.0)	44.9 (11.7)	25.0 (14.1, 35.9)	0.0002; *0.0001*
Children who received any brand of AL on the same day or following day of fever onset, at follow-up, by socio-economic status (wealth quintiles)^g^:				
Quintile 1 (most poor)	14.8 (20.6)	38.9 (18.3)	24.1 (4.6, 43.6)	
Quintile 2 (very poor)	16.6 (16.9)	40.0 (22.1)	23.4 (3.7, 43.0)	
Quintile 3 (poor)	16.6 (18.6)	50.8 (33.3)	34.2 (7.3, 61.2)	
Quintile 4 (less poor)	21.7 (18.6)	43.8 (22.4)	22.1 (1.5, 42.7)	
Quintile 5 (least poor)	15.4 (15.9)	47.8 (24.3)	32.4 (11.9, 52.9)	
Children who received Tibamal on the same day or following day of fever developing:				
Baseline	0 (0)	0 (0)		
Follow-up	0 (0)	29.7 (8.8)	29.7 (23.5, 35.9)	0.0001; *0.0001*
Children who received Tibamal on the same day or following day of fever developing at follow-up, by socioeconomic status (wealth quintiles)^g^:				
Quintile 1 (most poor)	0 (0)	30.1 (14.3)	30.1 (40.2, 20.0)	
Quintile 2 (very poor)	0 (0)	25.5 (19.9)	25.5 (39.6, 11.4)	
Quintile 3 (poor)	0 (0)	30.4 (21.3)	30.4 (45.4, 15.3)	
Quintile 4 (less poor)	0 (0)	32.5 (22.3)	32.5 (48.3, 16.8)	
Quintile 5 (least poor)	0 (0)	20.8 (22.1)	20.8 (36.4, 5.2)	

a Total number of children with fever in the previous two weeks present in the control arm: Baseline = 353; Follow-up = 344.

b Total number of children with fever in the previous two weeks present in the intervention arm: Baseline = 413; Follow-up = 417.

c *p*-Value: The *p*-value appearing first refers to the level of significance of the unadjusted difference between control and intervention arms at follow-up. The p value in italics refers to the level of significance of the adjusted difference between the control and intervention arm at follow-up.

d The reduced significance of the *p*-value after adjusting mainly reflects the significant negative relationship between baseline and follow-up values for this outcome. This negative relationship is likely to be caused by a tendency for those already using some kind of antimalarial at baseline to be more likely to start using Tibamal at follow-up (substituting one similarly priced product for another), as compared to those not using any antimalarial at baseline (for whom using Tibamal would represent an increase in average expenditure compared with their baseline purchases).

e Intraclass correlation coefficient control arm: Baseline: 0.009, follow-up: 0.02; intervention arm: baseline: 0.01; follow-up: 0.01 (based on formulae provided in [53]).

f Rank sum test: unadjusted analysis, p = 0.0013; adjusted analysis, *p* = 0.0003.

g Test for interaction between wealth quintiles and the intervention at follow-up: For the outcome “receiving any brand of AL on the same day or following day of fever developing,” *p* = 0.8749; for the outcome “receiving Tibamal on the same day or following day of fever developing,” *p* = 0.7445.

N, number of clusters.

**Table 3:** Actions taken for treating children aged 3–59 months with fever in the previous 2 weeks (a comparison of nine intervention and nine control clusters).

Care Sought	Control (N = 9) % (SD), n	Intervention (N = 9), % (SD), n	Difference in Means (95% CI)	*p*-Value^a^, Unadjusted; *Adjusted*
Government facility:				
Baseline	32.6 (12.6), 119	27.6 (14.9), 137		
Follow-up	36.4 (15.1), 118	29.0 (10.6), 116	−7.4 (5.7, −20.4)	0.2483; *0.1018*
Specialised drug store:				
Baseline	34.2 (12.9), 113	42.0 (13.1), 168		
Follow-up	23.8 (9.1), 78	30.4 (16.6), 121	6.6 (20.0, −6.8)	0.3140; *0.3642*
General store:				
Baseline	10.9 (5.2), 41	13.5 (5.2), 55		
Follow-up	20.3 (9.5), 67	27.2 (14.1), 115	6.8 (18.8, −5.1)	0.2442; *0.2158*
Missionary/private facility:				
Baseline	7.4 (4.8), 24	8.7 (7.5), 30		
Follow-up	9.3 (5.0), 30	5.4 (8.5), 19	−3.9 (3.0, −10.9)	0.2504; *0.3208*
Traditional healers:				
Baseline	0.5 (1.5), 1	0 (0), 0		
Follow-up	0.7 (1.3), 2	0.6 (1.9), 2	0 (1.6, −1.7)	0.9794; *0.9994*
Others^b^:				
Baseline	14.4 (5.8), 51	8.3 (7.3), 40		
Follow-up	9.5 (6.3), 31	7.2 (3.9), 29	−2.3 (2.9, −7.6)	0.3625; *0.6592*

a *p*-Value: The *p*-value appearing first refers to the level of significance of the unadjusted difference between control and intervention arms at follow-up. The *p*-value in italics refers to the level of significance of the adjusted difference between the control and intervention arm at follow-up.

b Others include: prayers, treatment with Western medications present at home, and treatment with home-made remedies.

n, Total number of visits; N, number of clusters.

### Antimalarials Obtained

There was an increase in children receiving antimalarial treatments from baseline to follow-up of 11.4% points in the control arm and 18.5% points in the intervention arm, with a significant difference at follow-up between the two arms (difference in means: 13.7%: 95% confidence interval [CI] 2.5, 24.9; unadjusted *p* = 0.0192; adjusted *p* = 0.0074) (Table 2).

The percentage of children receiving an antimalarial monotherapy (mainly amodiaquine, SP and quinine) fell by 7.0% points in the control arm and 26.6% points in the intervention arm (Table 2). At follow-up, the percentage of children receiving an antimalarial monotherapy in the intervention arm was lower than that in the control arm, although this was only of borderline significance in the adjusted analysis (difference in means: −10.5%: 95%CI: −3.9%, −16.9%; unadjusted *p* = 0.0036; adjusted *p* = 0.0518). Of those receiving monotherapies, few received an artemisinin monotherapy (an average of 1% at baseline and 0.2% at follow-up). The percentage receiving any brand of AL rose by 17.5% points in the control arm and 46% points in the intervention arm, and the percentage of children at follow-up receiving any brand of AL in the intervention arm was significantly greater than in the control (difference in means: 26.4%: 95%CI: 12.6%, 40.2%: unadjusted *p* = 0.0009; adjusted *p* = 0.0001) (Table 2). The increase in children receiving AL in the intervention arm was largely due to the uptake of Tibamal, which made up 63% of all AL received in this group. No caregivers reported purchasing Tibamal in the control arm. Of all those children who received any brand of AL, including Tibamal, a significant proportion received it either on the same day or following day of the fever developing (see Table 4 for results by cluster). The percentage of children receiving AL on the same day or the following day of the fever developing in the intervention arm at follow-up was significantly greater than in the control arm, with a difference between the arms of 25.0% points (95%CI: 14.1%, 35.9%; unadjusted *p* = 0.0002, adjusted *p* = 0.0001) (Table 2). This represents a substantial increase for this primary outcome, with the percentage of children receiving prompt AL treatment in the intervention arm being more than double that in the control arm at follow-up. There seemed to be no correlation between increasing wealth and the probability of receiving any brand of AL (*p* = 0.8749) or Tibamal (*p* = 0.7445) on the same day or following day of fever developing (Table 2, refer to footnotes). The variance observed between clusters was not large enough to warrant a weighted analysis (Table 4) [29]. Only 5.5% of homesteads had more than one child with fever in the past 2 weeks; allowing for homestead level clustering in the logistic regression did not affect the adjusted estimates (unpublished data).

**Table 4:** Percentage of children aged 3–59 months receiving any brand of AL on the same day or following day of fever onset, by cluster.

Cluster	Arm	Total No. of U5 Children, Baseline	Total No. of U5 Fevers, Baseline	Total No. of Fevers Treated with AL^a^ Same or Next Day, Baseline	% of U5 Treated with AL^a^ on Same or Next Day, Baseline	Total No. of U5 Children, Follow-Up	Total No. of U5 Fevers, follow-Up	Total no. of Fevers Treated with AL^a^ Same or Next Day, Follow-Up	% of U5 Treated with AL^a^ on Same or Next Day, Follow-up	Difference of Mean Between Baseline and Follow-Up
Akachachat	Control	116	46	2	4.3	104	27	8	29.6	25.3
Apokor	Control	146	55	1	1.8	144	50	9	18.0	16.2
Buchifi	Control	200	30	1	3.3	171	37	4	10.8	7.5
Kamunuoit	Control	135	30	2	6.7	149	28	7	25.0	18.3
Kanjala	Control	159	44	4	9.1	151	58	11	19.0	9.9
M.Central	Control	143	35	3	8.6	131	43	9	20.9	12.4
Musamba	Control	214	53	0	0.0	189	34	2	5.9	5.9
Nanderema	Control	156	43	4	9.3	156	42	16	38.1	28.8
Shianda	Control	112	17	1	5.9	110	25	3	12.0	6.1
Aludeka	Intervention	122	48	2	4.2	128	36	21	58.3	54.2
Eshibinga	Intervention	124	29	1	3.4	126	36	12	33.3	29.9
Kekalet	Intervention	143	45	2	4.4	149	51	25	49.0	44.6
Lunza	Intervention	151	25	2	8.0	157	35	10	28.6	20.6
Lupida	Intervention	166	68	7	10.3	166	79	34	43.0	32.7
Malaha	Intervention	187	36	0	0.0	195	30	9	30.0	30.0
Muyafwa	Intervention	183	61	5	8.2	170	58	30	51.7	43.5
Okatekok	Intervention	135	43	1	2.3	138	40	21	52.5	50.2
Sikinga	Intervention	157	58	1	1.7	128	52	30	57.7	56.0
**Total**		**2,749**	**766**	**39**		**2,662**	**761**	**261**		
Control total		1,381	353	18		1,305	344^b^	69		
Intervention total		1,368	413	21		1,357	417^b^	192		

a Refers to any brand of AL, including Tibamal.

b At follow-up, in the control arm five children had fever but information was missing on how the fever was treated; in the intervention arm eight children had missing details on whether fever was present within 2 weeks prior to the interview.

U5, age under 5 years.

**Figure 2:**
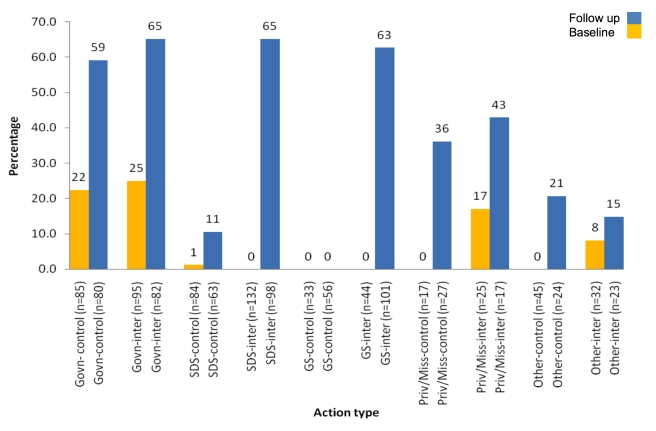
Percentage of visits to different sources of care at which any brand of AL was dispensed on the same day or following day of fever developing (a descriptive comparison between the nine intervention clusters and nine control clusters). Other includes treatment at home with home-made remedies or Western medication, traditional healers, or prayers. Standard deviations for each facility: Baseline control arm: government = 20; SDS = 4; GS = 0; priv/miss = 0; other = 0. Baseline intervention arm: government = 32; SDS = 0; GS = 0; priv/miss = 33; other = 10; Follow up control arm: government = 18; SDS = 20; GS = 0; priv/miss = 49; other = 36; Follow up intervention arm: government = 18; SDS = 21; GS = 25; priv/miss = 53; other = 34. Control, control arm; Govn, Government health facilities; GS, general stores; inter, intervention arm; Priv/Miss, private or mission health facilities; SDS, specialised drug stores.

We investigated the percentage of actions by source which resulted in any brand of AL being obtained on the same day or following day of fever developing (Figure 2), but did not assess the significance of difference between the arms at follow-up since the study was not powered for this subanalysis. AL dispensing at general stores increased from 0% to 63% from baseline to follow-up in the intervention arm, while no AL was dispensed in control arm outlets at baseline or follow-up. Similarly, in specialised drug stores, in the intervention arm AL dispensing increased by 65% points from baseline to follow-up (0% to 65%) compared to only a 10% point increase in the control arm (1% to 11%). Substantial increases were also seen at government facilities and private/mission facilities, but similar increases were observed in both arms (Figure 2).

### Accuracy of AL Doses Obtained and Consumed

Caregivers were asked to state the number of tablets they were provided with and the number their child consumed. Accuracy of dose obtained was defined as obtaining at least the correct number of tablets for their child's age. Accuracy of dose consumed was defined as reporting consumption of exactly the correct number of tablets for the child's age within 3 days of receiving the medication. We did not assess the precise timing of tablet consumption within this 3 day period due to the challenges of obtaining accurate recall.

Of all children receiving AL, just under 70% of children in both arms obtained an accurate dose at baseline (control 69.9% [SD: 33.8%]; intervention 68.6% [SD: 35.9%]), and just over 70% at follow-up (control 71.6% [SD: 20.9%]; intervention 76.9% [SD7.2%]). No significant difference was recorded at follow-up between the two arms (difference in mean 5.3%: 95% CI 20.9%, −10.3%; unadjusted *p* = 0.4836; adjusted *p* = 0.6545) (Table 5). Of all children obtaining AL, at baseline a correct dose was consumed by 40.5% (SD: 23.3%) in the control group and 53.1% (SD: 40.2%) in the intervention group. At follow-up this rose to 49.4% (SD: 24.8%) in the control arm and 67.0% (SD: 8.5%) in the intervention arm, but the difference was not significant at the 5% level (unadjusted *p* = 0.0606; adjusted *p* = 0.1095) (Table 5). In the intervention arm, 80.6% (SD: 9.6%) of caregivers received the correct dose of Tibamal for their child at follow-up compared to 70.7% (SD: 17.8) receiving the correct dose of any other brand of AL. Adherence to Tibamal at follow-up in the intervention arm was 71.8% (SD: 11.8%) compared to adherence to any other brand of AL at 61.1% (SD: 22.5%).

**Table 5:** Adequacy of AL doses obtained and consumed (mean of cluster summaries from nine intervention and nine control clusters).

Adequacy	Control^a^ (N = 9), % (SD)	Intervention^b^ (N = 9), % (SD)	Difference in Means (95% CI)	*p*-Value^c^, Unadjusted; *Adjusted*
Adequacy of dose obtained from the provider:				
Baseline	69.9 (33.8)	68.6 (35.9)		
Follow-up	71.6 (20.9)	76.9 (7.2)	5.3 (20.9, −10.3)	0.4836; *0.6545*
Adequacy of dose administered:				
Baseline	40.5 (23.3)	53.1 (40.2)		
Follow-up	49.4 (24.8)	67.0 (8.5)	17.6 (36.1, −0.9)	0.0606; *0.1095*

a Total number of doses in the control arm: Baseline = 26; Follow-up = 89.

b Total number of doses in the intervention arm: Baseline = 30; Follow-up = 221.

c *p*-Value: The *p*-value appearing first refers to the level of significance of the unadjusted difference between control and intervention arms at follow-up. The p value in italics refers to the level of significance of the adjusted difference between the control and intervention arm at follow-up.

N, number of clusters.

### Price Paid for Subsidised AL

95.3% (SD: 5.9%) of caregivers in the intervention arm at follow-up who bought Tibamal said they purchased it at the recommended retail price of 0.25 USD. Of those not paying this price, three paid less than 0.25 USD and five paid between 0.31 USD and 1.23 USD.

## Discussion

There has been considerable debate about how access to and quality of malaria treatment can be improved [3],[10],[11],[13],[14]. This study shows that a suite of ACT subsidies, retailer training, and community awareness activities can lead to substantial improvement in the uptake of prompt effective treatment for febrile children in rural Kenya. Although coverage still fell well below the 80% target set by the RBM, the percentage of children receiving AL during a fever episode in the intervention arm was more than double that in the control arm at follow-up, with more than half of those who received AL in the intervention arm receiving Tibamal, usually on the same day or the following day after fever onset. This was accompanied by lower use of antimalarial monotherapies at follow-up in the intervention group compared with the control group, although this difference was only of borderline significance. This is likely to have reflected “crowding out” of these antimalarials by the more effective subsidised AL. However, it may also have reflected government directives to phase out monotherapies such as amodiaquine at this time (personal communication with PPB and local amodiaquine manufacturer). In most cases, subsidised AL was purchased at the recommended retail price.

The increase in AL coverage observed does not seem to have resulted from a change in choice of providers, with treatment-seeking patterns remaining similar between the intervention and control arms. Instead, the intervention seems to have effected a change in the type of drugs dispensed in specialised drug and general retail outlets, with a major shift towards AL in both of these provider types.

It was notable that a substantial increase was also seen in AL coverage in the control arm between baseline and follow-up. This is likely to have reflected a reduction in AL stock-outs at government facilities between the two surveys in both arms. At baseline, public sector AL stock-outs were common, with only one third of facilities serving the study areas stocking both the 6- and 12-tablet packs of AL [20],[32]. At follow-up this figure had almost doubled to 65% [32]. This highlights that ensuring health facility AL stocks is also essential for improving AL access. Given that the study was carried out in the context of fluctuating supplies of AL at government facilities, it is possible that the increase in coverage from a subsidised retail sector intervention would be lower in a context with reliable public sector antimalarial supplies. However, it should be noted that government stock-outs of AL and other essential medicines are common in Kenya and other African countries, so this setting would not be considered atypical [20],[22],[33],[34].

In the intervention arm at follow-up, 77% of children receiving AL obtained an accurate dose, and 67% consumed the correct dose. No significant difference was observed in the accuracy of doses obtained or consumed between Tibamal (obtained only from retail outlets) and other AL brands (obtained mainly from government and private/mission facilities), although there was room for improvement in patient adherence to AL from both sources. In comparison, a 2005 review looking at adherence in the community to chloroquine, which also has a 3 day regimen, showed only a median of one third using it correctly [35]. Other studies on ACT adherence have shown varying results, ranging from 39% to 90% [36]–[39], though the higher figures obtained in some studies may reflect study designs where caretakers were aware that their compliance would be monitored. There are a number of limitations to the measurement of adherence used here and in similar studies. It may be difficult for caregivers to recall such details over a 2 week period, or they may deliberately misreport tablet consumption if they are concerned about revealing inappropriate dosing. Also, in formal health structures such as government health facilities the child's weight as opposed to age may be used to determine the dose [40], so children who did not fall into the standard weight range for their age may have only seemed to have obtained the wrong number of tablets. However, there are several reasons why adherence may truly have been suboptimal, including poor knowledge of dosing regimens, lack of advice from providers, and stock-outs of one of the AL pack sizes meaning that children may have been sold an inappropriate pack for their age. During focus group discussions, caregivers also reported stopping medication as soon as the fever subsided, and believing that the child's recovery would hasten if the tablets were given at more frequent intervals than stipulated in the dosing regimen [41]. Interventions to improve adherence could include reducing stock-outs of specific pack sizes, encouraging shopkeepers to talk through the package dosing instructions with caretakers, and the use of mass media to emphasise the importance of completing the full dose [35].

Only one suspected ADR was reported through the retailer referral forms for a child who had recently taken Tibamal. The child was experiencing vomiting, shivering, and refusing to eat or drink, and was referred to the nearest government health facility. It was unclear whether the lack of other reported referrals reflected a genuine lack of potential ADRs or a failure to report them. During focus group discussions, caregivers and shopkeepers commented that children who suffered any suspected ADRs from Tibamal, or who did not get better, went directly to formal health facilities, without going back to the retail outlets, meaning that retailer referral forms may be inappropriate and/or unnecessary for monitoring pharmacovigilance under retail distribution.

Other studies evaluating the effectiveness of distributing subsidised ACTs through the private retail sector have shown mixed findings. In pilot projects in Tanzania and Uganda there was a rapid uptake of subsidised ACTs and a decrease in the use of antimalarial monotherapies, with good adherence to target retail prices [8],[9],[12]. By contrast, in Cambodia and Senegal, availability of subsidised ACTs remained irregular, which was associated with retail prices above the target level in Cambodia but not in Senegal [12]. No other published data are yet available on the impact of private sector ACT subsidies on coverage of prompt effective treatment, and robust data on other strategies to improve ACT coverage are limited [11]. There is however evidence that provision of ACT through community medicine distributors (CMDs) could also lead to high levels of ACT coverage, with a multicountry study in Ghana, Nigeria, and Uganda finding that 59% of children reporting fever in the past 2 weeks had received ACT from a CMD [42].

Several reviews have documented the challenges of drawing firm conclusions about strategies to improve retail sector treatment provision due to the limitations of existing studies, which often lack adequate controls [10],[11],[43]–[45]. We selected a cluster randomized design to significantly reduce the influence of chance, bias, or confounding due for example to variations in public sector drug stocks, weather patterns, and malaria awareness campaigns [46]–[49]. While such randomized controlled trials are argued to have high internal validity, there is concern that they may lack external validity because the study design demands implementation practices that would be unrealistic in operational settings. In this study, implementation was relatively typical of routine practices, without the insistence on “ideal” delivery and adherence required in clinical efficacy trials. However, the need to avoid contamination of control sublocations meant that drug delivery and consumer education had to be modified from standard practices. The implications of this are discussed further below, where we consider likely differences between the Tibamal intervention, and the AMF-m.

A number of other potential weaknesses in the study should be highlighted. The analysis was carried out as two separate cross-sectional surveys and did not adjust for children who may have had fever at both survey time points. This may have resulted in an underestimation of the primary outcome; however, we believe that any possible underestimation as a result should be relatively small. A limited degree of clustering occurred within homesteads within survey rounds, but this did not affect the estimates. Contamination is an important risk in study designs of this kind, and we therefore investigated the exposure of households in the control arm to the intervention. No children in the control arm were reported to have received Tibamal at follow-up (Table 2). In addition, at follow-up 82% of caregivers in the intervention arm had heard of Tibamal, compared to only 7% in the control arm [26].

The comparison of the suite of interventions with the control does not allow us to isolate the contribution of each component. However, we consider this appropriate given the consensus in the literature that interventions of this kind need to be multifaceted, incorporating both consumer- and provider-focused strategies [10],[50],[51].

Care should be taken in extrapolating or generalising these findings. This study was undertaken in three districts, all within one province in Kenya, and was restricted to rural areas, so the generalisability of the results to other areas should be carefully considered. In some respects these districts can be considered relatively representative of Kenya as a whole. For example, the 2 week fever prevalence, ITN use, and education levels reported in this study are similar to those reported in national surveys [52]. 58% of households in the control arm and 60% in the intervention arm were classified as poor, compared to a national average of 54% [18]. However, this area has very high levels of malaria endemicity compared with the rest of the country, and a relatively active retail drugs market, with many specialised drug stores. Although treatment-seeking patterns for fever in Kenya can be considered relatively typical of sub-Saharan Africa, there are important variations between countries in the share of treatment sought in the retail sector and the nature of retail outlets providing drugs [4],[10]. Since follow-up data were collected only 8 months after Tibamal distribution began and 4 months after the start of community awareness activities, it is not known if Tibamal uptake would stabilise or increase as consumers and providers become more familiar with the medication over time.

In addition, there are a number of differences between this pilot and the planned AMF-m roll-out, meaning that the results should be used with caution for predicting AMF-m impact. This intervention was targeted at children aged 3–59 months only, but under AMF-m subsidised drugs will be available to all age groups. Under AMF-m subsidised drugs will be distributed through existing private and public sector distribution chains. By contrast, in this pilot Tibamal was distributed directly to retail outlets in order to avoid contamination of the control arm; it is possible that use of existing private sector distribution chains may either improve or worsen retail sector availability, and the likely impact on final retail prices is unclear. No mass media promotion was used in the pilot, again to avoid contamination, though this could be a major feature of AMF-m roll out, potentially enhancing community awareness of AL availability and dosing. Finally, this pilot included all medicine retailers including general stores; however, most countries planning to implement AMF-m intend to restrict the availability of subsidised AL to registered pharmacies and in some cases drug stores. It is unclear how such a narrower range of retail outlets will affect both uptake and adherence.

A number of key questions around ACT subsidy programmes remain unanswered, above and beyond those of generalisability and differences between this intervention design and that proposed by AMF-m as described above. As noted above, even in the intervention arm, the coverage of prompt ACT treatment of 44.9% remained well below the 80% RBM target, so the need to identify additional strategies to increase coverage remains. A key priority is improving accessibility in the public sector by strengthening drug supply and reducing unofficial user fees. As around a third of fevers are currently treated at public facilities, increasing ACT dispensing to these cases has the potential to have a major impact on treatment coverage. For those patients who find public facilities inaccessible and even subsidized drugs in the retail sector too expensive, it may be necessary to consider other community-based strategies such as the use of CMDs. Moreover, the retail sector intervention itself could have been further strengthened by the use of mass media for promotion (not feasible during this study due to the cluster-randomised design), stronger enforcement of the monotherapy ban by the government, reduction in Tibamal stock-outs, and/or training of a higher proportion of retailers on Tibamal (a requirement for outlets stocking the product). There were several potential reasons for the relatively low proportion of outlets in the intervention area reporting trained staff (43%) at follow-up. Some outlets identified for training were unable to attend due to other commitments, or were closed when training invitations were distributed. Others did not meet the eligibility requirements for training at baseline (functioning for a minimum of 6 months and selling an antimalarial or antipyretic within the past year) but did meet these at follow-up, and many new outlets appeared to have opened up, leading to an increase in eligible outlets of 74 in the control arm and 126 in the intervention arm between baseline and follow-up (Figure 1). This increase may have been as a result of field workers becoming better at locating outlets, or it could simply reflect the fluidity of the retail sector. All trained shops were given the option of stocking AL but not all did so, with most blaming insufficient funds. These issues highlight the challenges of maintaining a trained cadre of AL retailers in such a dynamic market.

There is concern that such strategies to increase retail sector coverage could lead to substantial increases in overtreatment, because many of those seeking ACTs in the private sector will not be parasitaemic. As coverage increases, research is urgently needed to assess how enhanced diagnosis—for example through rapid diagnostic tests—can be implemented in the private retail sector to limit use of antimalarial treatment to confirmed cases of malaria. There is also concern about the capacity for appropriate pharmacovigilance when ACTs are distributed more widely outside formal facilities, and a need to evaluate strategies to improve adherence to ACTs obtained from all sources. Finally, the cost and cost-effectiveness of subsidy programmes should be calculated and compared with other public sector and community-based strategies for improving malaria treatment and prevention.

## Supporting Information

Figure S1 Map of Kenya displaying district boundaries and malaria classifications.
(0.24 MB DOC)

Figure S2 Maps of study districts showing control (orange) and intervention (green) sublocations.
(0.71 MB DOC)

Text S1 Trial protocol.
(1.25 MB PDF)

Text S2 CONSORT checklist.
(0.22 MB DOC)

## Abbreviations

ACT: artemisinin-based combination therapy
ADR: adverse drug reaction
AL: artemether–lumefantrine
AMF-m: Affordable Medicines Facility-malaria
CI: confidence interval
CMD: community medicine distributor
EA: enumeration area
ITN: insecticide-treated net
PCA: principal components analysis
PPB: Pharmacy and Poisons Board
PSI: Population Services International
SD: standard deviation
SP: sulphadoxine–pyrimethamine
