# Supplementary material for: The Impact of Retail-Sector Delivery of Artemether–Lumefantrine on Malaria Treatment of Children under Five in Kenya: A Cluster Randomized Controlled Trial
Source: PLoS Med. 2011 May 31;8(5):e1000437. doi: 10.1371/journal.pmed.1000437 (PMC3104978; doi:10.1371/journal.pmed.1000437)
Supplement: Figure S1 — Map of Kenya displaying district boundaries and malaria classifications. (0.24 MB DOC) [file pmed.1000437.s001.doc]

**Figure S1: Map of Kenya displaying district boundaries and malaria classifications.** Butere/ Mumias, Teso and Busia are the chosen sites for the pilot.

|  | 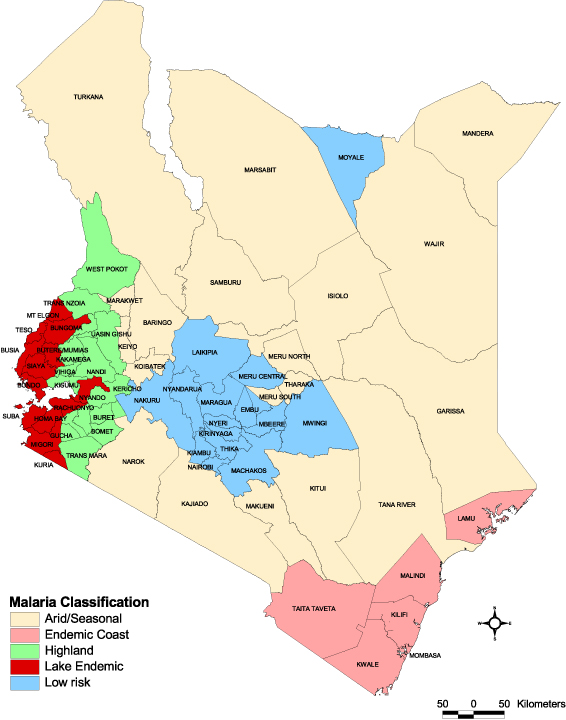 |
| --- | --- |
| **Teso**  **Busia**  **Butere/ Mumias** |
|  |
