# Supplementary material for: The Impact of Retail-Sector Delivery of Artemether–Lumefantrine on Malaria Treatment of Children under Five in Kenya: A Cluster Randomized Controlled Trial
Source: PLoS Med. 2011 May 31;8(5):e1000437. doi: 10.1371/journal.pmed.1000437 (PMC3104978; doi:10.1371/journal.pmed.1000437)

**Figure S2: Maps of study districts showing control (orange) and intervention (green) sub locations.** Figure 2a Busia District; Figure 2b Teso District; Figure 2c Butere Mumias District.

*N.B: ‘Other’ (see Legend) refers to all sub locations that do not fit the sub location criteria (e.g. urban or peri-urban and with populations <2,500 and > 10,000).*

**Figure S2a**


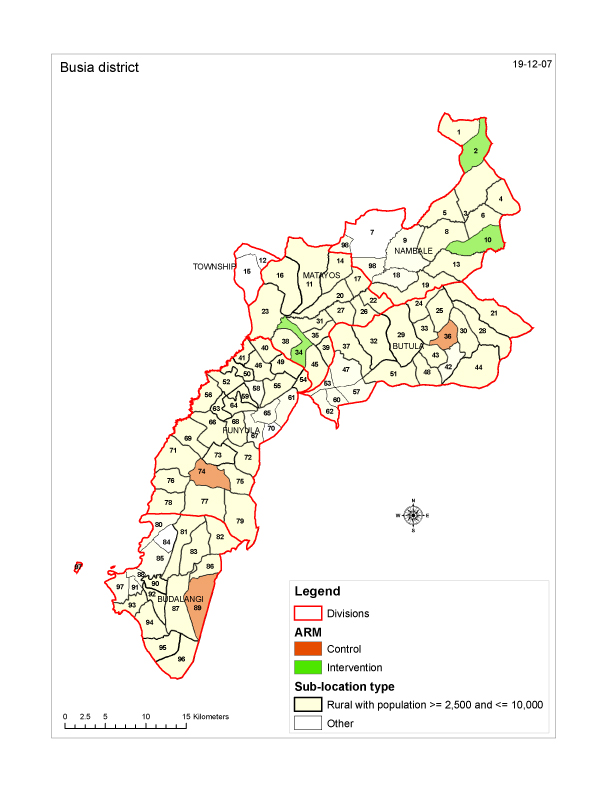


**Figure S2b**


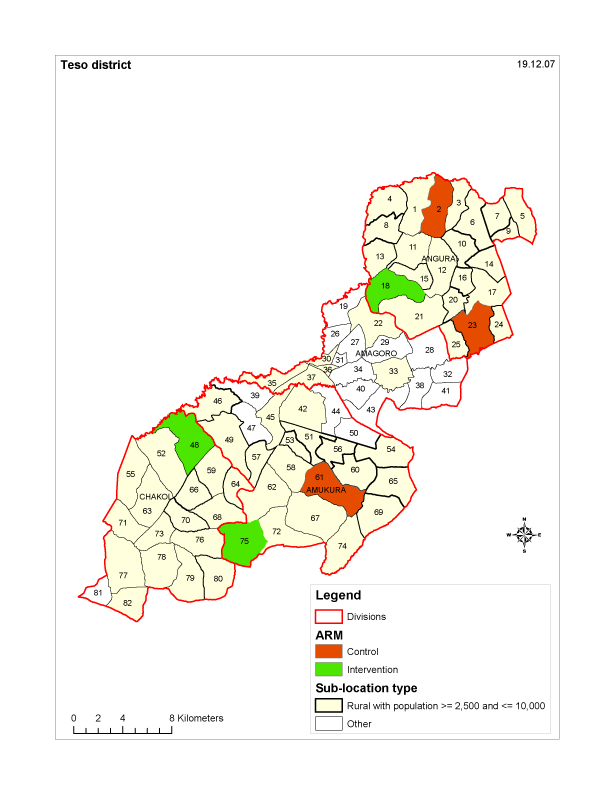


**Figure S2c**


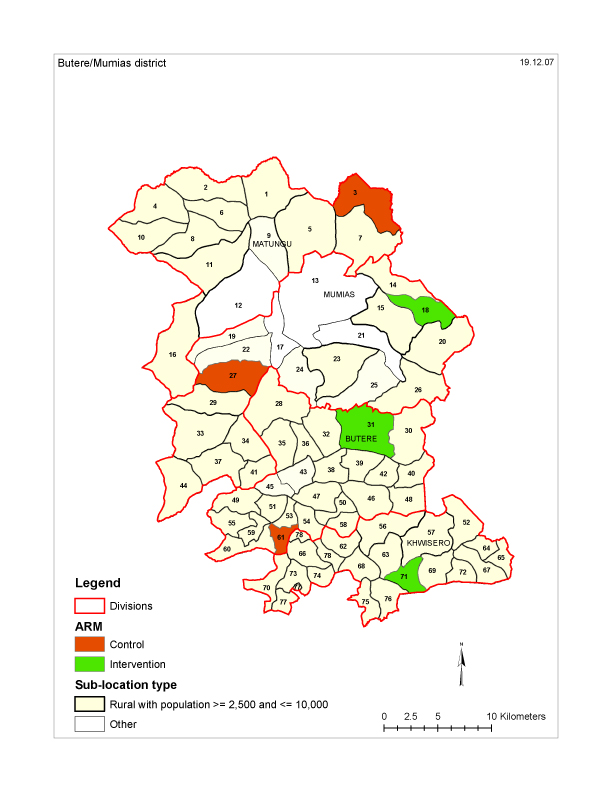

Supplement: Figure S2 — Maps of study districts showing control (orange) and intervention (green) sublocations. (0.71 MB DOC) [file pmed.1000437.s002.doc]
