# Supplementary material for: The Impact of Retail-Sector Delivery of Artemether–Lumefantrine on Malaria Treatment of Children under Five in Kenya: A Cluster Randomized Controlled Trial
Source: PLoS Med. 2011 May 31;8(5):e1000437. doi: 10.1371/journal.pmed.1000437 (PMC3104978; doi:10.1371/journal.pmed.1000437)
Supplement: Text S1 — Trial protocol. (1.25 MB PDF) [file pmed.1000437.s003.pdf]

1. Title of the Project

The Impact of Retail Sector Delivery of Artemether-Lumefantrine on Effective Malaria Treatment of Children under five in Kenya

2. Investigators and Institutional Affiliations

Principal Investigator
Miss BB Kangwana[1]

Co-investigators
Dr C Goodman[1]
Dr G Fegan[1]
Dr AM Noor[1]
Dr AJ Nyandigisi[2*]
Dr WS Akhwale[2*]
Dr Jayesh Pandit[3*]
Prof RW Snow[1]

1) KEMRI / Wellcome Trust Research Programme (KWTRP), Centre for Geographic Medicine Research- Coast, P.O. Box 43640, 00100 GPO, Nairobi, Kenya

2) Division of Malaria Control, Ministry of Health, Government of Kenya, P.O. BOX 20750, Nairobi, Kenya

3) Department of Pharmacovigilance, Pharmacy and Poisons Board, Ministry of Health, Government of Kenya, P.O. BOX 27663-00506, Nairobi, Kenya

3. Abstract

In 2006 Kenya began implementing its new anti-malarial treatment policy that replaced sulphadoxine/ sulfalene-pyrimethamine (SP) with a more efficacious artemisinin-based combination therapy (ACT), artemether-lumefantrine (AL). The treatment is being distributed through the public sector free of charge but the level of access to AL remains low. The aim of this study is to evaluate to what extent the provision of pre-packed, subsidized AL delivered through private sector retailers will increase the proportion of children under five, with fever, receiving appropriate anti-malarial treatment. The intervention will be implemented by the Division of Malaria Control (DOMC) in collaboration with Population Services International (PSI). KEMRI/ Wellcome Trust Research Program (KWTRP) will be responsible for the evaluation of the intervention. The effectiveness of this intervention will be evaluated through a pre-post cluster randomised controlled trial. Baseline data will be collected before the intervention and follow up data 9 months after the start of the intervention from both households and retail outlets. The outcomes monitored will be derived from a list of key indicators approved by the DOMC as information they require to inform their policies on increasing access to ACTs. The data will be collected using six data collection activities: 1) Retail census, 2) Household survey, 3) Provider survey, 4) Mystery shopper, 5) Focus group discussions, and 6)

Documentation of context. The results from this study will support the DOMC in national strategic planning for improving access to effective malaria treatment.

4. Introduction

a) Malaria Background

Malaria remains an important health problem with more than 3 billion individuals living at risk of the disease [1]. It is estimated that 300 to 660 million cases are caused by the most virulent of the plasmodium species, *Plasmodium falciparum* [2]. *P. falciparum* contributes to 90% of the malaria burden in Africa and 1 million childhood deaths per year are a direct consequence of the parasitic infection [2, 3]. In Kenya, approximately 20 million people live in areas that expose them to the risk of developing malaria. By 1997, it was estimated that 145,000 children aged between 0-4 years were admitted to hospital from this disease annually and 26,000 died [4]. Although there has been a recent decline in cases, the numbers remain unacceptably high [5].

The Roll Back Malaria (RBM) Global Partnership was created in 1998 to increase international awareness of malaria and rally support for the control of the disease [6]. One of its four core targets is that '80% of those suffering from malaria should receive appropriate treatment within 24 hours by 2010' [7]. The rapidly increasing resistance to widely used and inexpensive anti-malarials, as well as the inability of the health care sector to provide sufficient services to all those who need care have created barriers to achieving this goal in many parts of Africa, including Kenya [8, 9].

b) The Policy Change in Kenya

Artemisinin derivatives used in combination with other effective anti-malarial treatments are currently considered very effective, with cure rates of over 90% [10]. They have been shown to be well tolerated and to lower transmission rates within communities by reducing gametocyte loads. It is thought that the rate of development of resistance to this treatment will be greatly reduced because of the short half life of artemisinin and its use in combination with other treatments [11, 12]. To date, 56 countries have incorporated ACTs into their malaria treatment guidelines [13].

Due to a precipitous decline in its clinical efficacy SP was replaced with AL for the treatment of uncomplicated malaria in Kenya [14, 15]. Kenya changed its anti-malarial treatment policy in 2004 with public sector distribution of AL beginning mid 2006. The policy change process in Kenya was to occur in phases over a five year period with the first two years seeing AL distributed free of charge through public facilities. This would allow time for the country to develop experience before the policy could be rolled out to a wider range of providers such as private-for profit clinics and the retail sector in order to increase access [14, 15].

c) Treatment of Malaria within the Public and Private Sector

After more than a year of distributing AL free of charge within the public sector, studies carried out in Kenya revealed that only 26% of children presenting with fever in public health facilities who would benefit from this treatment were prescribed it. This is despite interventions such as in-service training and awareness campaigns implemented to promote uptake [16]. This poor adherence to guidelines is consistent with findings in other parts of Africa, such as Zambia where

after one year of policy implementation in the public sector, AL was only prescribed for 22% of febrile children [17-22]. According to a recent evaluation of the Kenyan public sector, weak product supply chains, poor training and supervision as well as a lack in health care workers' prescribing confidence are all thought to have contributed to poor prescribing practices [23]. Weaknesses within the public sector have been acknowledged by the government who are working in collaboration with both local and international organisations to improve performance [24].

Even if a majority of children accessing care within the public sector received AL, the treatment seeking behaviour patterns in Kenya are such that a significant proportion of healthcare is first sought through the private sector [25-28] (see typology of health care providers in Table 1).

Table 1: Health Care Providers in Kenya – a Typology

Sector	Definition	Constitutes
Public	Providers funded by and in direct control of the government	• Government health care facilities<br>• Community Owned Resource Persons
Private	Providers who fall outside the direct control of and are not funded by the government	• Not for profit (Mission and Non-governmental organization) health care facilities and community owned resource persons<br>• Private/ commercial health care facilities<br>• **Retailers: registered pharmacies, general provision shops and mobile hawkers**<br>• Traditional healers and herbalists

A household survey carried out in four endemic districts in Kenya revealed that 90% of caregivers took some action to treat a child's fever within 48 hours of symptom onset. Of these, 47% first sought treatment in the private retail sector and only 35% went to public or not for profit health facilities [27]. A small proportion, 23.3% of all these fevers were treated with an anti-malarial within 48 hours, of which 61% were obtained from the public sector, 28% from the retail sector and only 10% by self administration of medicines available in the household. The proportion of febrile children who received first line recommended AL within 48 hours was only 10.2%. As expected, the majority of AL (95%) was dispensed from public health facilities [27]. What this demonstrates is that health care for malaria is heavily sourced from the private retail sector; however, the services received tend to be poor. Care provided from this sector for the treatment of malaria is mainly based on ineffective medications [25-27]. Since a high proportion of individuals seek treatment within the retail sector [29-31], encouraging AL distribution within this sector at an affordable price, along with improving the quality of health care services offered is expected to significantly expand the coverage of effective malaria treatment within the community [32].

d) Improving Delivery of Anti Malarials through Retailers

Home Management of Malaria (HMM) is a strategy that has been supported by RBM with the aim of increasing prompt and effective treatment of malaria within the community. This strategy exploits the strengths and improves the services offered by providers outside the public facilities.

It can be delivered through retailers, community health workers or other community members and is generally implemented alongside public sector facility delivery [6, 31, 33-52].

HMM in the retail sector can be broken down into various intervention components which include training and capacity building; demand generation/ consumer information; quality assurance and the creation of an enabling environment. Previous pilots carried out on HMM interventions within the retail sector show that they are more successful if they begin with a comprehensive situation analysis to understand the legal and market environment of the retail sector; if they involve all the necessary stakeholders from the retailers to public health officials; if the strategy consists of a combination of approaches; and if the interventions are on-going to ensure sustainability of outcomes [6, 31, 33-51].

Studies have been carried out to evaluate the value of HMM within the retail sector however, the evidence remains limited. A number of HMM interventions based on ACTs are currently underway in Tanzania, Uganda, Rwanda, Cambodia, Madagascar and Nigeria. Preliminary results from the ACT pilot in Tanzania indicate that delivery of ACT through retailers increased uptake of the medication, but access of the medication to those in lower socio economic quintiles remained poor (unpublished data from the Clinton Foundation Tanzania Pilot ACT Study, 2007). However, no evaluations have yet been completed and none are yet underway in Kenya.

Previous HMM studies have focused on other anti-malarial therapies such as SP, however the nature of the dosing and cost of ACTs may alter the outcomes of the interventions. Moreover, many evaluations focus on intermediate outcomes such as provider knowledge and behaviour rather than more health related outcomes such as medicine use within the community. In addition, the outcomes measured vary between studies making comparability difficult [32, 52, 53]. No studies have used a cluster randomised approach, and most studies do not even include a control group, relying instead on pre and post data only [32, 53]. This may have exposed studies to possible confounders. Since very few studies have evaluated outcomes by socio-economic status, there is little information on how equitable these interventions are [32]. Also, many studies have evaluated outcomes soon after implementation of the interventions (3-4 months) so the sustainability of these programs and their long term effectiveness remain unknown [32, 53]. There has only been one comprehensive cost-effectiveness analysis of HMM carried out in Kenya [54], therefore little is known on how much the government should budget to roll out HMM interventions on a national scale and the relative value for money of these strategies. Lastly, very few pilots have been published in peer reviewed journals, thus the quality of the data available may be questionable [32, 53].

5. Justification for Study

Kenya has planned to roll out AL into the private sector in 2009 [14] however this decision should be based on sound evidence. Even with the information available from the previous monotherapy retail sector interventions, many key questions regarding HMM interventions and the distribution of AL through the private retail sector remain unanswered. These include a) can retailers provide AL in an appropriate manner, and b) will HMM interventions within the private retail sector significantly improve access to AL in Kenya, and in an equitable way. It is hoped that this and other HMM pilots, based on common outcome measures will be carried out in Kenya to address these questions and augment the data that are already available. All these data

can then be collated to provide substantial evidence that can be used by policy makers to guide their decisions on if and how to distribute AL outside of public facilities.

6. Null Hypothesis

The provision of pre-packaged, subsidized, AL delivered through private sector retailers will have no effect on improving the coverage of prompt effective anti-malarial treatment.

7. Objectives

7a) General Objectives

To evaluate to what extent the provision of pre packaged, subsidized, AL delivered through private sector retailers will improve the coverage of prompt effective anti-malarial treatment.

7b) Specific Objectives

1) To determine the impact on the proportion of children under five with fever being treated promptly with appropriate anti-malarial treatment, and adhering to the correct dose (***accessibility and utilisation***).

2) To determine if private sector retailers can deliver AL to appropriate standards of quality for the treatment of fever in children under five years (***provision***).

3) To determine distribution of benefits of retail sector delivery of AL by socio- economic status (***equitable coverage***).

4) To explore reasons for the impact observed and identify any challenges in the implementation process (***explanation of experience***).

8. Design and Methodology

General Study Design

The study design employs a pre-post randomised cluster controlled design, with clusters (sub locations) randomly allocated to intervention and control groups[1]. 9 intervention and 9 control sub locations will be located across 3 districts in Western province. In the intervention areas, pre-packed, subsidized paediatric AL will be introduced in September 2008 through selected private sector retailers serving the sub location population, together with a range of supportive activities (see below). Public sector delivery will continue as normal. In the control sub locations, routine delivery of AL through public sector outlets will also continue as normal. Control and intervention areas will be selected with consideration of geographic proximity to ensure that the control areas are not 'contaminated' by the intervention. Baseline data collection will take place

[1] The effectiveness of the intervention will be measured as the difference in differences between the baseline and follow-up surveys in the intervention and control groups.

in July-August 2008 and the post intervention data collection will occur in July-August 2009. These survey dates are considered to coincide with peak malaria season and therefore have been selected to maximise the number of fevers identified.

The Intervention Package

The intervention package will be designed and implemented by the DOMC in collaboration with Population Services International (PSI), Ministry of Health (MOH) staff at the province and district level and other key stakeholders. The role of KEMRI/ Wellcome Trust Research Program (KWTRP) will be limited to evaluation. PSI is a non profit organisation that promotes healthy behaviour through the use of health marketing programs. Its main focus is on malaria, family planning, HIV/AIDS/STI prevention, diarrhoeal diseases, micronutrient deficiencies and waterborne disease. PSI will draw from their previous experiences from the delivery of retail sector ACT in a number of countries including Tanzania, Rwanda, Cambodia, Madagascar and Nigeria. A brief summary of the intervention is described below. A more detailed intervention plan will be developed in collaboration with the above stakeholders.

The Product – Pre-packaged AL for children under 5: PSI and the DOMC will develop a branded pre-packaged AL product for the treatment of malaria in children. In line with dosing recommendations, two doses will be developed; one for 6 months to 3 year olds (5-15kg) and one for 4 to 8 year olds (15-25kg). The target group for this intervention will be children under five years of age. The product's outer packaging and insert will use locally developed low literacy instructions to improve appropriate dose recognition by caregivers and shopkeepers and promote adherence to the full regimen. The process of product development will be based on extensive formative research, pre-testing and modification in consultation with the case management team of the DOMC. The product's instructions will also include details on the Integrated Management of Childhood Illness (IMCI) danger signs and the need to refer to the public health service severe conditions and children under one year[2].

Price: It is proposed that the consumer price of the pre-packaged AL will be competitive with the currently available SP and amodiaquine (AQ) brands of malaria treatment. This price is envisaged to be approximately 20 Kenya Shillings (Ksh) per treatment course for all under five doses. This approach will ensure that price is not a significant barrier to accessing effective anti-malarial treatment and that there is no significant price incentive for consumers to choose an inappropriate malaria treatment. This price will also undercut artemisinin monotherapies substantially meaning that we can expect demand for them to be reduced in the intervention sites. The price will be clearly shown on the packaging and will be widely promoted in communications to encourage adherence.

Place/Distribution: The pre-packaged product will be distributed directly to selected retail outlets on a routine basis. Outlets selected will be those that are perceived to be well established, respected businesses by the local community. These outlets should already stock anti-malarials

[2] Note that whilst the AL formulation is suitable for children under 1 year but over 5kg, the proposed program will promote all under 1's to be referred to the nearest health facility. This is because of the increased risk of progression to severe malaria and hence, vulnerability of this age group. Guidelines will be included in the packaging for under ones, directing how to use the medicine in an emergency, so long as the child is over 5kg, so that treatment can commence, buying time until professional medical care can be sought.

or anti-pyretics and have been functioning for a specified minimum amount of time (to be defined following initial retail census). Selected outlets will have a notice visible to consumers informing them that the pre-packaged AL can be purchased from the store, which will tie into promotional activities.

Shopkeeper training: All selected shopkeepers will be given brief focused training. Shopkeepers will be trained to ask key questions related to symptoms and age of the child which will form the basis of their response in terms of referral to a public health facility or supply of an appropriate prepackaged dose of AL, combined with key messages on administering the treatment and adhering to the regimen. The content of the shopkeeper training will be based upon the experiences of the extensive shopkeeper training studies already undertaken in Kenya by the Ministry of Health (MOH) and KEMRI/ Wellcome Trust Research Programme (KWTRP) [55, 56]. Shopkeepers will be trained on appropriate storage conditions of AL as well as identifying IMCI danger signs, and the need to refer specific conditions to public health facilities. The trainings will integrate local nurses as technical advisors. PSI will develop training, point of sale materials, and job aids including simple treatment algorithms with guidance from the DOMC and the drug policy technical working group (DPTWG) to improve the quality and quantity of information provided to consumers.

Promotion: PSI will carry out a series of promotional activities in the intervention areas and related dominant market centres. Messages will target caregivers of children under five and will promote appropriate treatment seeking behaviour including the benefits of AL and its availability both in public sector facilities and identified private sector outlets. Messages will be delivered through a range of interpersonal communications media including community drama, road shows, chief's *barazas*, and community group educational sessions. MOH public health technicians will also be briefed on the program and encouraged to conduct community awareness programs and integrate messages into health talks. In addition, where there are active district ITN advocacy/ information, education and communication (IEC) working groups, their support will be sought in conducting awareness raising in the targeted communities. These activities will be supported by point of sale materials, posters, leaflets and localized media such as wall paintings (mass media strategies will not be used in this pilot phase to avoid contamination between intervention and control areas).

Diagnosis: Diagnosis of malaria within the retail outlets will be based on a history of fever, as is standard practice in a majority of endemic rural public health facilities and stated in the World Health Organisation (WHO) guidelines [57, 58]. Shopkeepers will be educated on this through training and IEC material.

Drug Regulation: AL is currently registered in Kenya as a prescription only medicine (POM). However, the transitional plan for implementation of the ACT Malaria Treatment Policy in Kenya states that AL should eventually be deregulated and that studies must be conducted to guide this process. PSI, KWTRP and the DOMC will seek exemption from the POM regulation for this pilot.

Pharmacovigilance: The Pharmacy and Poisons Board (PPB) have developed guidelines and tools for the collection of pharmacovigilance data on AL since its release in the public sector. The intervention package will therefore be implemented in collaboration with the PPB to ensure

that pharmacovigilance requirements are met. Shopkeepers will be educated on possible adverse effects and will advise caregivers to seek care from the nearest health facility for any suspected adverse drug reactions (ADRs). The shopkeeper will complete a CHW referral form for each such caregiver; one copy will be taken by the caregiver to the facility and the other copy will remain with the shopkeeper and later collected and sent to the PBB. All ADRs seen within health facilities will be reported back to the PPB. The PPB along with district investigation teams will be involved in following up any serious ADRs. The DOMC and PSI will work together with the PPB and provincial pharmacist to ensure staff in health care facilities within the province receive training on pharmacovigilance with regards to AL and to make them aware of the pilot.

8a) Study Sites

The study will take place in rural areas of 3 districts in Western Province. This province was selected for the following reasons:

- It is malaria endemic (*Figure 1*),
- PSI has a strong team of permanent sales staff on the ground[3],
- There are no other planned HMM interventions[4],
- Presence of a relatively active retail market.

Within Western province, Bungoma district was excluded because of the extent of previous malaria-related interventions which may make it atypical. Mount Elgon district was excluded because of the current political insecurity in that area. The districts chosen for the pilot were therefore Butere/ Mumias, Teso and Busia (*Figure 1*). Butere and Mumias were officially divided into two districts in mid 2007, but because this split is recent, the two districts will be treated as one for the purposes of this study.

The pilot intervention will be implemented at the sub-location level. The sub-location was selected for the following reasons:

- To provide a reasonable scale for implementation,
- To ensure no contamination between intervention and control (which would be more likely if larger areas were used),
- To contain the total medicine cost for the pilot phase.

The following inclusion criteria were used for selecting intervention and control sub-locations:

- They are rural, as this is where access to health care is most limited and so HMM most needed. In addition retailers in urban and peri-urban sub-locations serve customers from a much wider area, which would lead to contamination;
- The populations within the sub-locations are between 2,500 to 10,000; smaller sub-locations were excluded to ensure there was a reasonable scale for implementation and adequate

[3] PSI staff in Coast Province are much more limited, especially in rural areas.
[4] The Great Lakes University plan to implement an HMM intervention using community health workers in Nyanza Province.

sample sizes for the evaluation; larger sub-locations were excluded to contain the cost of the pilot.

Through random selection 18 sub-locations were identified, stratified across the 3 districts, each district having 3 controls and 3 interventions (*Figure 2-4*). To avoid contamination a buffer of at least 2 sub-locations was maintained between any selected sub-location. The sub locations shown below are the outcomes from our preliminary sample selection. The final selection will be confirmed after further visits to the areas by PSI to investigate feasibility.

Figure 1: Map of Kenya displaying district boundaries and malaria classifications. Butere/ Mumias, Teso and Busia are the chosen sites for the pilot.

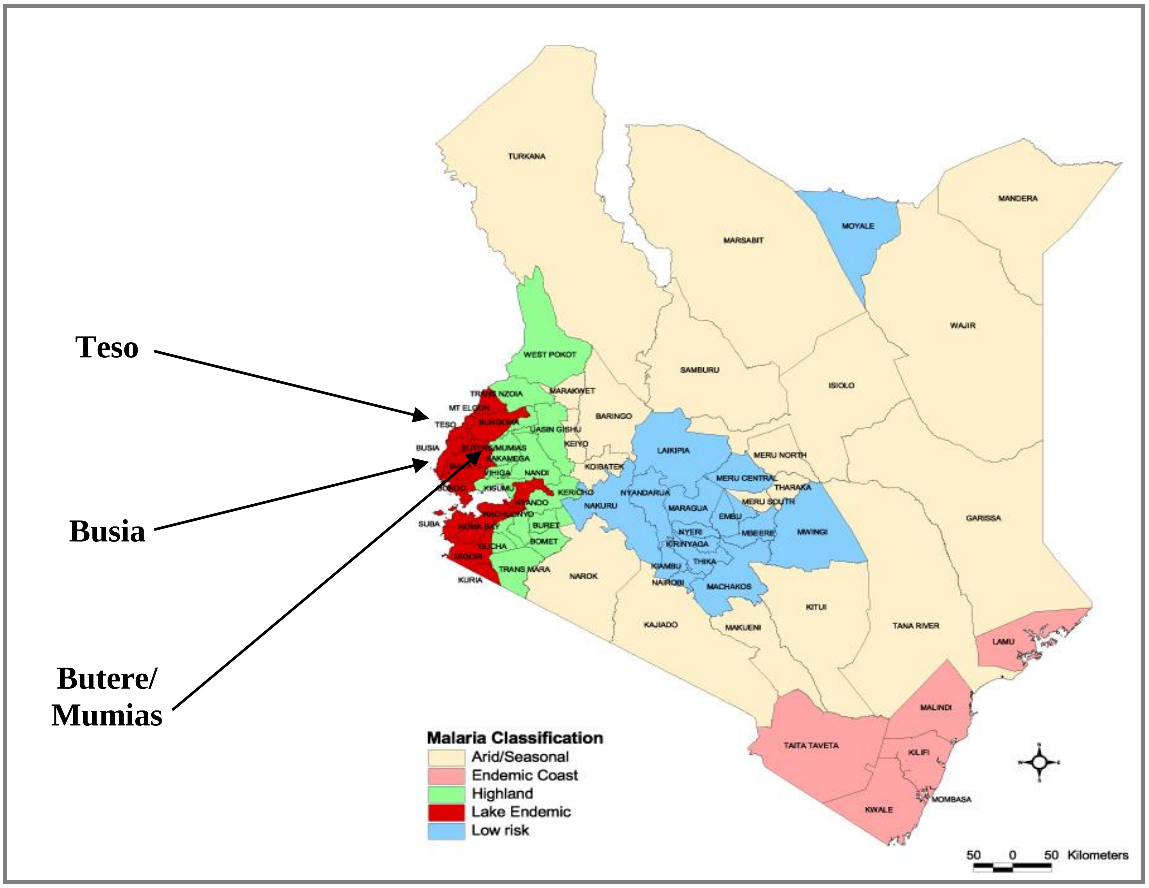

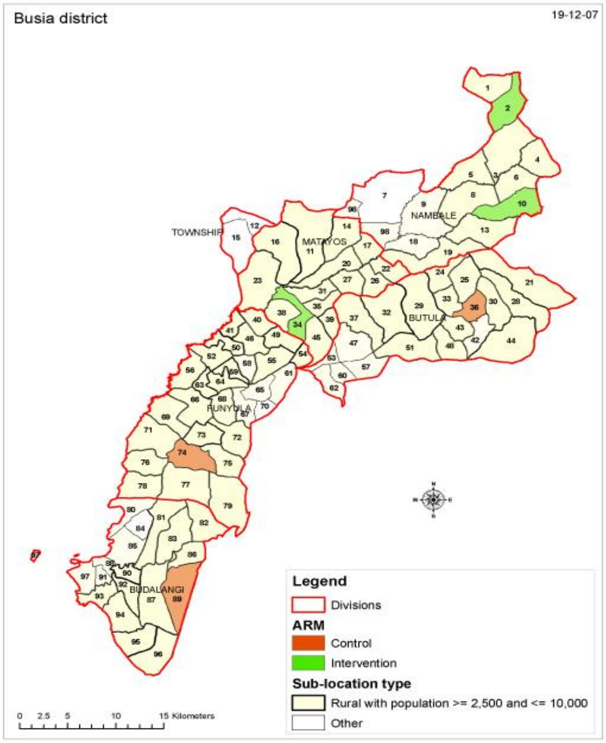

Figure 2 (left): Map of Busia district showing control (orange) and intervention (green) sub locations.

N.B: ‘Other’ (see Legend) refers to all sub locations that do not fit the sub location criteria (e.g. urban or peri-urban and with populations <2,500 and > 10,000).

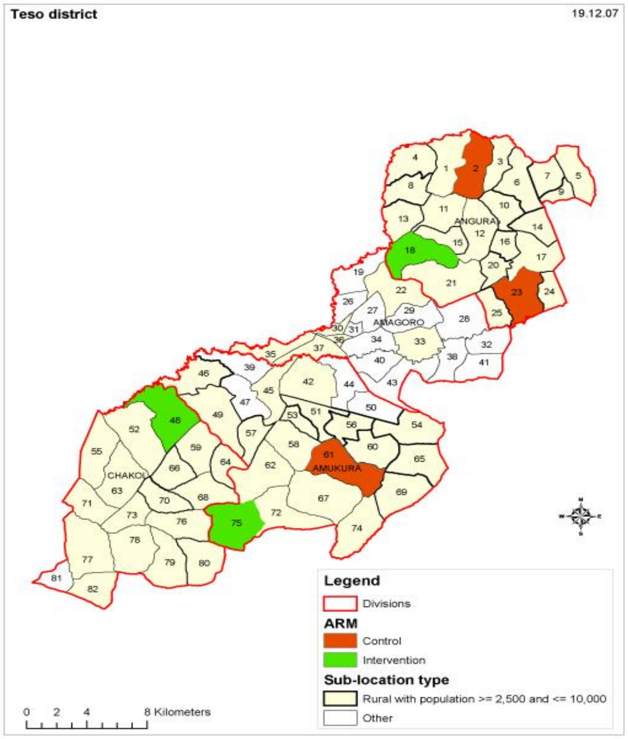

Figure 3 (left): Map of Teso district showing control (orange) and intervention (green) sub locations.

N.B: ‘Other’ (see Legend) refers to all sub locations that do not fit the sub location criteria (e.g. urban or peri-urban and with populations <2,500 and > 10,000).

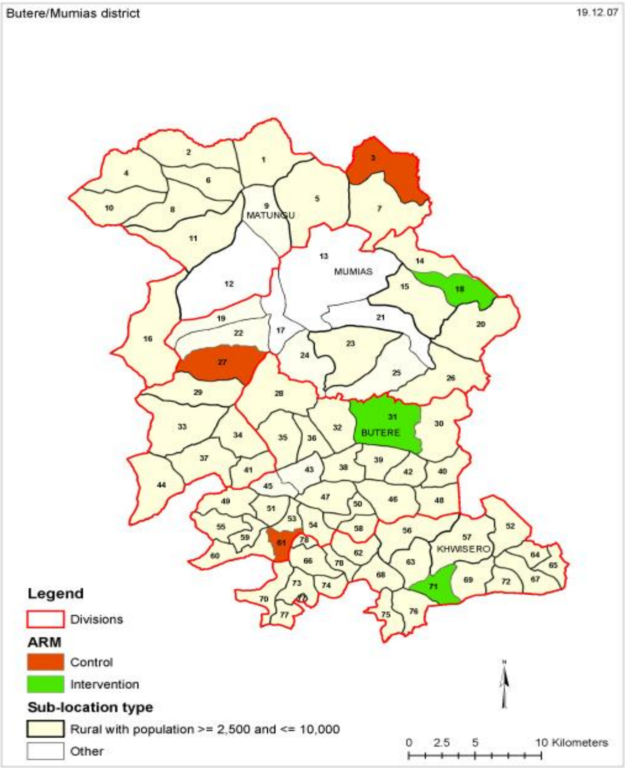

Figure 4 (left): Map of Butere/ Mumias district showing control (orange) and intervention (green) sub locations.

N.B: 'Other' (see Legend) refers to all sub locations that do not fit the sub location criteria (e.g. urban or peri-urban and with populations <2,500 and > 10,000).

Table 2: District Demographics

	BUSIA	TESO	BUTERE/ MUMIAS
NO. OF SUB LOCATIONS	99	83	79
% OF SUB LOCATIONS RURAL	76	66	75
% HOUSEHOLD HEADS COMPLETED PRIMARY SCHOOL	57	54	58
NO OF HEALTH CARE FACILITES*	39	21	51
%POOR (RANGE ACROSS SUB LOCATIONS)	67 (53-74)	50 (44-68)	62 (53-73)
EST POP 2007 (AVERAGE PER SUB LOCATION)	370,608 (4,964)	227,058 (2,769)	573,275 (7,350)
POP_ DENSITY/ KM^2	433	406	611

* These include Ministry of Health and other ministries, mission and non-governmental health facilities

Table 2 gives an overview of the districts' demographics. Teso is the least poor district with 50% of the population living under the poverty line, followed by Butere/ Mumias, with Busia being the least well off. Butere/ Mumias is the district with the largest population, Teso is the least populated, containing around half the population of Butere/ Mumias. Butere/ Mumias is also the most densely populated district with 611 people per KM^2. The number of health care facilities are greatest in Butere/ Mumias followed by Busia and then Teso. In all three districts just over 50% of household heads have completed primary school. The languages spoken in Busia and

Butere/ Mumias include Luo, Kiswahili and different dialects of Luhya, and in Teso, Luhya, Kiteso and Kiswahili.

Previous malaria control initiatives such as training of shopkeepers and community awareness programmes have been carried out in Busia by both the Ministry of Health and Non Governmental Organisations (NGOs). The DOMC and PSI will be intervening in all these districts to implement malaria community awareness programs and train public facility health care staff, funded by the President's Malaria Initiative (PMI). These activities will cover both pilot intervention and control areas, and take place after baseline data collection for this study has been completed. This allows us to assess the value added of retail sector provision once appropriate efforts to strengthen the public sector have been put in place. A more detailed context analysis will take place during the pilot to identify other health initiatives within the districts that may affect the outcomes of the pilot.

Table 3: Demographics for the selected sub locations

SUB_LOCATION	DISTRICT	%POOR	UNIQUE_ID^	ESTPOP2007	POP_ DENSITY/ KM^2	ARM
MAGOMBE CENTRAL	BUSIA	64	89	3575	200	Control
KANJALA	BUSIA	68	36	2703	389	Control
NANDEREMA	BUSIA	66	74	3490	298	Control
MUYAFWA	BUSIA	65	34	4053	473	Intervention
LUPIDA	BUSIA	68	2	4418	328	Intervention
SIKINGA	BUSIA	69	10	5945	392	Intervention
AKACHACHATA	TESO	48	23	2626	293	Control
APOKOR(ANGURAI)	TESO	51	2	3185	374	Control
KAMUNUOIT	TESO	49	61	3273	297	Control
ALUDEKA	TESO	48	48	3275	285	Intervention
OKATEKOK	TESO	52	75	3955	375	Intervention
KAKALET	TESO	49	18	3370	372	Intervention
SHIANDA(BM)	BUTERE/MUMIAS	58	61	3030	748	Control
BUCHIFI	BUTERE/MUMIAS	61	27	8659	574	Control
MUSAMBA	BUTERE/MUMIAS	62	3	8079	476	Control
ESHIBINGA	BUTERE/MUMIAS	69	71	4134	643	Intervention
LUNZA	BUTERE/MUMIAS	61	31	9294	482	Intervention
MALAHA(BM)	BUTERE/MUMIAS	63	18	6094	612	Intervention

^Represents the numbers assigned to the sub locations on the district maps

Table 3 shows the demographics for the selected sub-location populations, the unique ID represents the numbers of the individual sub-location on the district maps (*Figures 2-4*). These data reflect the experience at the sub location level. The populations within the selected Teso sub-locations tend to be less poor than the other two districts, with the percentage living below the poverty line ranging from 48-52%. In Busia the percentage living under the poverty line ranges from 64-69% while in Butere Mumias it is from 58 to 69%. Butere Mumias is the most densely populated with sub-location population densities ranging from 476 to 748 per KM^2. Busia and Teso's population densities are quite similar with Teso ranging from 293-374 per KM^2 and Busia from 200 to 473 per KM^2. Across the three districts, the average % poor and population densities between the control and intervention sub locations are similar (*Table 4*).

Table 4: Comparison of Percentage Poor and Population Density between Intervention and Control Sub Locations, across all three Districts.

ARM	AVERAGE %POOR	AVERAGE POP_ DENSITY/ KM2
CONTROL SUB LOCATIONS	58	4291
INTERVENTION SUB LOCATIONS	60	4949

Retail outlets can either be found within a market centre where there is a group of shops together or outside of market centres as ‘stand alone’ or individual shops. There are on average 8 retail outlets in a market centre. Based on study visits and discussions with PSI and MOH staff, it is estimated that an average of 8-10 ‘stand alone’ retail outlets and 3-4 market centres containing retail outlets sell medicines to a rural sub-location in Busia; in Butere/ Mumias around 15-18 ‘stand alone’ retail outlets and 5-6 market centres, while in Teso it is about 12-14 ‘stand alone’ retail outlets and 5-6 market centres. These details will be confirmed by further visits by PSI and through the initial retail census (see below) to be carried out by KWTRP.

8b) Study Populations

A total of six data collection activities will be conducted to evaluate the intervention. Specific study populations for each activity are listed below.

i) Criteria for inclusion of subjects: see under specific surveys below

ii) Criteria for exclusion of subjects: see under specific surveys below

iii) Rationale for animal use and justification for animal species chosen: not applicable

8c) Sampling

i) Sampling size determinations: see under specific surveys below

ii) Sampling procedures: see under specific surveys below

8d) Procedures

i) Description of the type of data to be collected and the collection procedures to be followed

A list of key indicators for all pilots has been developed in collaboration with the DOMC, and were approved by them (*Appendix 4*). They have been divided into compulsory and optional indicators. The indicators have been identified through consideration of relevant DOMC targets; Global Fund indicators; Roll Back Malaria monitoring and evaluation reference group indicators; and Global ACT subsidy monitoring and evaluation frameworks. The compulsory indicators are to be monitored by all pilots evaluating interventions to increase anti-malarial access outside the

public sector within Kenya. Standardizing outcome measures between pilots will allow for data to be compared across studies, and will also ensure that the studies provide the DOMC with the relevant information they require for policy implications. The Red Cross will also run an HMM pilot based on these indicators, other future pilots are to be developed.

In this study we will focus on the compulsory indicators and some optional indicators that we consider to be relevant to monitoring the effect of the intervention. As previously mentioned, these indicators will be monitored through 6 data collection activities, each with its own data collection tool (*Figure 5*).

Figure: 5: Data Collection Activities

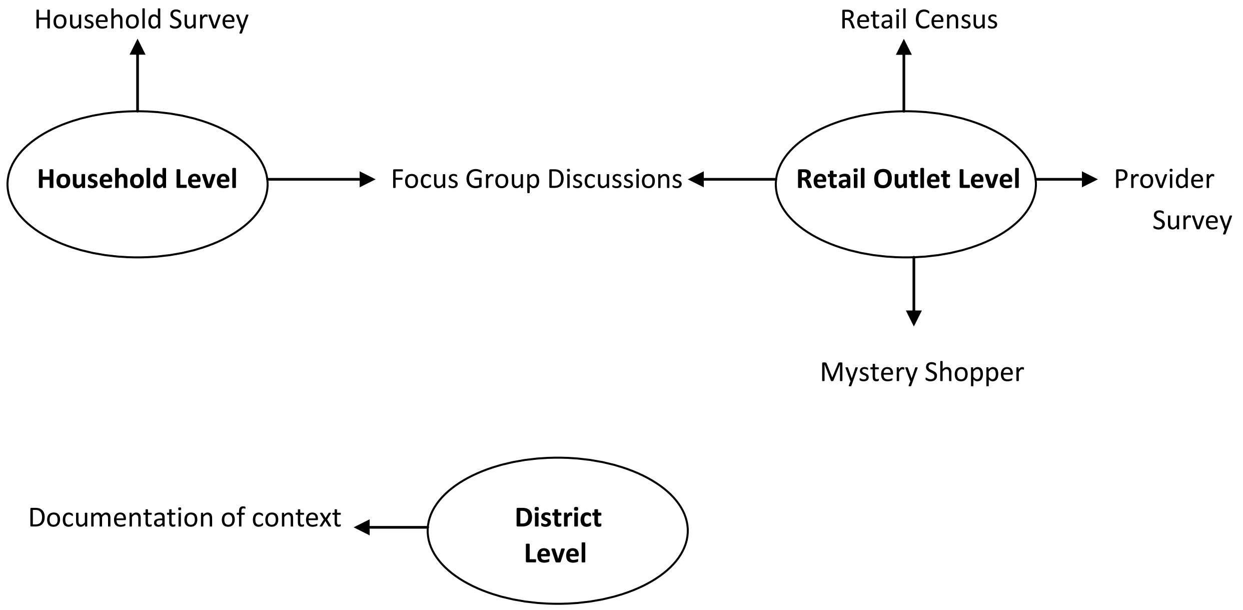

The tools for each activity have drawn on studies which have evaluated similar outcomes within the retail sector and the household level [27, 60-62]. The tools will be piloted before the studies to ensure questions are interpreted correctly by the respondents. The household survey, retail census, provider survey and mystery shopper survey will be administered both before and after the interventions in both the intervention and control areas. The focus group discussions will only be administered post intervention and only in the intervention areas. Data collection for the documentation of context will be on-going throughout the evaluation.

1) Retail Census Survey:

The retail census aims to identify all retail outlets selling medicines that serve the population of the study sub-locations. This will include retail outlets located both within the study sub-locations and those in neighbouring sub-locations that are frequently used by the study population. For the purposes of this study, retail outlets include all shops selling medicines, including general retailers and registered pharmacies where these exist. Outlets such as bars, hardware shops and salons are excluded. Retail outlets will be identified initially from lists

provided by PSI. This will be supplemented by visiting the areas and confirming the list with local leaders. In addition, a snowballing technique will be used where shopkeepers of known retail outlets will be asked to identify other retailers within the local area. All identified retail outlets being regularly accessed by the populations within the selected control and intervention sub locations will then be mapped. Field workers will visit each retail outlet and position it using a Global Positioning System (GPS) unit (Garmin etrex (Garmin Ltd, Kansas, USA) and Trimble 12 band). Three longitudinal and latitudinal readings will be taken for each outlet and the average reading noted to minimise errors in positioning. Current estimates indicate that the accuracy of GPS readings is within 15 meters of the true position [63]. Details of the type of anti-malarial medicines stocked within each outlet will also be recorded.

The database derived from these analyses will form the sampling frame for other retail outlet surveys and will also be used to monitor any leakage of public sector AL into retail outlets, any stocking of project AL by untrained private retail outlets, to monitor the coverage of the intervention and the distance of retail outlets to neighbouring households, hence accessibility. The following indicators will therefore be monitored from this survey:

A. The proportion of sub-locations with at least one AL source

B. Availability of public sector AL in private retail outlets

C. Availability of private sector project AL in untrained private retail outlets

D. The proportion of retailers stocking project AL at follow up survey

The retail census will take place before other baseline data collection activities and will be updated before the follow up data collection activities both in the control and intervention areas. Data will be collected on retail census questionnaires. It is estimated that the census will cover a maximum of 49 retail outlets per sub location; 882 retail outlets in total (*Table 5*). Verbal consent to collect these data will be obtained from the available shopkeeper in each retail outlet.

2) Household Survey

The indicators to be measured include:

A. The proportion of children under 5 years with fever in the past 2 weeks who started treatment with AL within 24 and 48 hours of fever onset, overall, by socio-economic group (SEG) and treatment source

B. The proportion of children under 5 years with fever taking AL who adhered to the treatment dose, by source

C. The proportion of under 5 years with fever who took an anti-malarial monotherapy in the past 2 weeks

D. The proportion of children under 5 years with fever in the past 2 weeks who sought treatment by source (e.g. public, mission & commercial health facilities, pharmacies, other retail outlets, Community Owned Resource Persons (CORPs), traditional healers and other sources)

E. The proportion of non-target household members receiving intervention AL to treat a fever within the past two weeks[5]

F. Household cost of fever episode (for all completed episodes)

G. The proportion of households within 30 minutes and within 1 hour travel time from an AL source

H. The proportion of caregivers with knowledge of malaria symptoms, danger signs, AL and correct AL dose for a 4 year old

These indicators aim to monitor any changes in access to effective anti-malarial treatment, adherence, treatment seeking behaviour and knowledge of malaria within the households. Indicators B, C and H will also be important as factors which potentially affect the development of resistance.

The household survey will be carried out both at baseline and post intervention, within the intervention and control sub-locations. Three enumeration areas (EAs) will be selected per sub-location (with probability proportional to size of EA). We will randomly sample 20 homesteads within each EA, equivalent to a total of 540 homesteads (roughly 1922 households) in each group. We will aim to visit the same 20 homesteads in the follow-up as in the baseline to optimise statistical power. Where it is not possible to visit the same homestead we will replace it with its nearest non-sampled neighbour. The sampling frame of homesteads in each EA will be created with the help of local village leaders. The list will be confirmed by visiting the EAs a month before the field work commences and recording GPS co-ordinates for each identified HH.

A household survey questionnaire will be administered to the household head and all care givers of children under 5, filling in one questionnaire per child. All indicators apart from indicator E will be focused on children under five years since they are the most vulnerable group. To monitor '*the proportion of non targeted household members receiving the intervention AL*' (indicator E), a brief additional questionnaire will be administered to all members of the household aged 5 or over who had a fever within the past 2 weeks (care givers will be interviewed on behalf of children aged 5-15), to estimate what treatment was used and from which source. Indicator D will provide information on the leakage of project AL to untrained outlets. This will augment data collected for indicator C in the retail census i.e. '*availability of private sector project AL in untrained private retail outlets*'.

[5] As the pack sizes cover children aged 6 months to 3 years and 4 to 8 years, we will assess the proportion of people aged 9 years and over who obtained intervention AL. In addition, as the main target group for the interveniton is under fives, we will assess the proportion of people aged 5 years and over who obtained intervention AL.

The survey is restricted to fevers occurring in the previous two weeks since the recall period beyond this time point is questionable [64]. Although the RBM indicator states that treatment should be sought within 24 hours, 48 hours has also been included in indicators A and B as this could still be considered prompt. The denominator for 24 and 48 hours in these indicators includes all individuals reporting fever and visited by interviewers 2 to 3 days or more after the onset of symptoms. As the intervention aims that all under 1s should be referred to a health facility, and should not therefore receive AL from retailers except in an emergency, Indicator A will be calculated both with and without this group in the numerator. For indicator B, adherence will be defined as taking the quantity of medicine specified in the MOH treatment guidelines, and both under and over dosing will be considered as non-adherence. The timing of administration between doses within the 3 days will not be considered as recall of specific times may prove difficult. A photo-illustrated guide will be used to aid in the identification of anti-malarial treatments [25, 64].

Wealth indicators based on those used by the Demographic and Health Surveys (DHS) will be collected this survey to determine the socio-economic status of each household and gain better information on the equity impact of the intervention [65, 66].Wealth indicators will include housing quality, sources of income, education status and ownership of livestock of the household head. Principal components analysis (PCA) will be used to construct a household wealth asset index from the information collected. The derived wealth indices will then be used to classify households into wealth quintiles [67]. In addition households can be linked to national socio-economic quintiles using asset weights from nationwide surveys. Wealth indicators will be collected during both baseline and follow-up household surveys since they are subject to variation [67]. GPS co-ordinates will also be taken for each household interviewed to determine the distance of the household to the nearest retail outlet and facility.

Written consent will be obtained from the household head or their representative and verbal consent from the interviewee. The village elder or chief will be informed about the survey in advance. The village elder will be asked to aid the field team in identifying the households to be interviewed within the designated enumeration areas.

Sample Size Determination for Household Survey

The sample size calculation is based on a cluster randomized before and after study design, with 9 intervention and 9 control sub locations stratified across the three districts (Busia, Teso, Butere/ Mumias). The key outcome indicator is indicator A: *The proportion of children under 5 years with fever in the past 2 weeks who started treatment with AL within 24 and 48 hours of fever onset, overall, by SEG and treatment source*. The study design chosen for this study is a cluster randomised sample, for logistical reasons. The cluster randomisation will occur at the EA level with the primary sampling unit being the household.

Using a 'difference in proportion' sample size calculation and an estimated design effect the following was calculated (*Appendix 3*):

To detect a difference of 20 percentage points in the key outcome indicator, with a 5% significance and 80% power:

$n = [(z_{alpha/2} + z_{beta})^2(p1\ (1-p1) + p2(1-p2)] / (p1 - p2)^2$

Where:

n = desired sample size (recent childhood fevers) in each group

$z_{alpha/2}$ = 1.96 (5% significance)

z_{beta} = 0.84 (80% power)

p1 (control) = 0.2, the starting percentage (data derived from a fever survey carried out in June 2007 showed 11% of children under the age of five had access to an anti-malarial within 48 hours. This was increased to 20% to be conservative as a larger p1 will require a larger sample size).

p2 (intervention) = 0.4, the starting percentage plus a 20% point increase (assuming that a 20% point increase is the minimum necessary to justify the importance of the intervention in public health policy terms).

n = 79 childhood fevers required in each group (i.e. control and intervention arms)

Based on previous surveys carried out in Kisii, Kwale and Bondo [27, 60], 3.4 homesteads (HS) need to be visited to find one recent childhood fever. Therefore a total of 269 HS will be required in each group.

Assuming a design effect of 2 to allow for clustering, a total of 538 HS will be required in each group, equivalent to 60 HS per sub-location (or around 214 households per sub-location [27, 60]). If 20 HS were surveyed per EA, we would need to evaluate 3 EAs per sub-location. This would equate to a total of 27 EAs in the intervention group and 27 in the comparison[6]. This will be feasible for the majority of EAs, as data from Kisii, Kwale and Bondo show that the number of HS per EA ranges from 3-110 (mean=36, inter quartile range= 23-41). Where EAs with less than 20 HS are selected, they will be replaced with the nearest EA with at least 20 HS (*Table 5).*

In addition, sample size calculations estimates were also made using the Hayes and Bennett Cluster Sampling Formula IJE 1999 [7] (*Appendix 2*), which also showed that 27 EAs in each group would be adequate as a sampling frame.

---

[6] If the design effect is increased to 3, 45 EAs will need to be evaluated in the intervention group and 45 in the control group.

[7] Hayes RJ, Bennett S. Simple sample size calculation for cluster-randomized trials. International Journal of Epidemiology, Vol 28, 319-326.

3) Provider Survey

The indicators to be measured include:

A. The median price charged by retail outlets for AL by age band

B. The proportion of retail outlets with no expired AL available in stock

C. The proportion of retail outlets reporting stock outs of AL within the past 2 weeks

D. The proportion of retail outlets storing AL appropriately (see definition of 'appropriately' below)

E. The proportion of retail outlets that have copies of the materials/ job aids required by the intervention (e.g. leaflets, posters, guidelines, pharmacovigilance reporting forms)

F. The proportion of retail outlet staff who know the correct information about malaria diagnosis and treatment practices

These indicators aim to monitor the affordability of anti-malarial therapies within the retail sector, whether any subsidies are passed onto the consumer and the nature of retail supplies of anti-malarials and source. In indicator C, the period of monitoring stock outs i.e. two weeks is chosen for recall purposes. In indicator D, 'appropriate' storage refers to keeping medicines off the floor, in a dry area, away from direct sunlight, and with the packaging intact. Also included in this survey will be questions to assess the knowledge of the provider including what he/ she knows about malaria symptoms, diagnosis, treatment and ADRs.

The indicators will be assessed through a provider survey questionnaire. Questions will be asked to the retailer present at the shop. Verbal consent to administer this questionnaire will be sought from the interviewees.

Sample Size for the Provider Survey: The survey will be conducted in outlets selected to deliver the subsidised ACT product. Depending on the total number of outlets included in the intervention, we may include all outlets in the provider survey, or take a random sample. It is estimated that around 150 outlets will be surveyed in each group (i.e. control and intervention groups (*Table 5*).

4) Mystery Shopper Survey

The indicators to be measured include:

A. The proportion of retail outlet staff that dispense AL to patients presenting with fever

B. The proportion of retail outlet staff that dispense the correct dose of AL to patients presenting with fever

C. The proportion of retail outlets that provide appropriate information to caregivers on how to give/ take AL

D. The proportion of encounters where retail outlet staff asked for one or more IMCI danger signs to determine need for referral

E. The proportion of retail outlet staff who told caregivers about any signs of progressive illness and recommended a referral visit to a facility or clinic if the signs appear

F. The median price charged for an AL dose by age group

The aim of this survey is to analyze the patient provider interaction to give better information on actual rather than self-reported provider behaviour. This survey will take place before the provider survey to avoid making retailers aware that they are being evaluated. It will augment and allow for triangulation with the provider survey.

The survey will take the form of a mystery shopper survey. Here, field workers will pose as ordinary care givers seeking care for a four year old child with fever. Questions will be administered to the staff selling medicines at that time, and any recommended medicines will be purchased by the field worker. Details of the interview will be filled in away from the outlet. Questions will be standardized between retail outlets to allow for comparisons of behaviour between retailers and over time. The indicators and data collection tools for this activity will be further refined once a detailed outline of the intervention activities have been developed. This method was chosen instead of direct observation or exit interviews because it minimises any potential bias that may occur through knowing one is being observed. In addition, achieving a reasonable sample size for exit interviews could be very time consuming in outlets which receive very few fever customers per day. This technique does however raise some ethical concerns as informed consent cannot be obtained from the medicine seller at the time of the interview [68-70]. Verbal consent will therefore be sought from all shopkeepers during the retail census for their willingness in principle to participate in the mystery shopper survey. They will be informed on what the survey involves, however neither whether their shop will be selected for the survey nor the date of the visitation will be revealed as this may affect the study outcomes.

Sample Size for Mystery Shopper Survey: The mystery shopper survey will be conducted in the same outlets as the provider survey (*Table 5*).

5) Focus Group Discussions (FGDs)

Focus Group Discussions will be carried out only after the implementation of the intervention and only within the intervention sub locations. The purpose is to ascertain the perceptions and opinions of the intervention from community members and shopkeepers. This may help confirm and explain observations in the quantitative data. Each group will contain 6 to 10 people. Two FGDs will take place per intervention sub location (i.e. a total of 18 FGDs, see *Table 5*). In each sub location a group of selected shopkeepers and a group of selected caregivers of children under five will be interviewed separately. To ensure that a range of experiences are presented,

shopkeepers will be selected purposively, based on data from the provider survey to ensure that they include a mixture of those considered to be well and poorly performing. Care givers will be purposively selected based on treatment seeking behaviours, as revealed from the household survey.

Discussions will take the form of semi-structured interviews. Shopkeepers will be asked about the benefits and costs of being involved in the program as well as problems they may have experienced in the implementation process. Care givers will be asked about their opinions on treatment sources, availability of products, barriers faced in seeking treatment and any concerns they may have regarding the program. In addition the use of intervention AL in adults will also be explored (drug misuse).

A letter of invitation to the FGD sessions will be presented to the care-giver, his/ her household head and to the shopkeepers to be interviewed. It will explain the duration of the interview and that they may need to arrange for someone to carry out their daily activities for that period of time. A travel allowance will be given to each interviewee and drinks will be provided during the sessions. Verbal consent to carry out these discussions will be gained from the interviewees prior to initiation of the discussions. Discussions will be tape recorded and records supplemented by notes taken during the discussions by a field assistant. This will ensure the capture of verbal and non verbal cues and facilitate exploration of arising issues.

6) Documentation of Context

We will undertake careful documentation of context at national and district level and other factors which may have influenced the study outcomes in both the intervention and control areas. A list of issues that may be of concern will be prepared to provide a framework for data collections. Throughout the study, a series of desk-work analyses of newspaper articles, minutes to meetings, draft proposals, budget allocations and memos, as well as in-depth discussions with the District Health Management Teams (DHMTS) at the local level, the DOMC at the national level and other relevant bodies will take place. From this, a chronology of events will be documented, as well as a summary of the events, the locations and the duration. These data will be taken into consideration during evaluation of the study outcomes.

Table 5: Summary of Sample Sizes Required for each Data Collection Activity

DATA COLLECTION ACTIVITY	UNIT OF ANALYSIS	NUMBER PER SUB LOCATION	TOTAL NUMBER OF SUB LOCATIONS	TOTAL SAMPLE SIZE IN ALL SUB LOCATIONS
RETAIL CENSUS	Retail outlet	49	18	882
HOUSEHOLD SURVEY	Homestead (household)	60 (214)	18	1080 (3845)
PROVIDER SURVEY	Retail outlet	17	18	306
MYSTERY SHOPPER	Retail outlet	17	18	306
FOCUS GROUP DISCUSSIONS	Shop keeper groups of 6-10	1	9	9
FOCUS GROUP DISCUSSIONS	Care giver groups of 6-10	1	9	9

Estimating the Cost and Cost-effectiveness of the Intervention

Cost and cost-effectiveness data is considered important by the DOMC for assessing the affordability and value for money of all potential interventions. However, it is recognised that the unit costs of small scale pilots will not reflect those of nation-wide implementation, as they exclude certain activities (e.g. mass media) and do not reflect economies of scale. KWTRP will not therefore conduct a detailed costing of the pilot. However, the DOMC have decided to employ a costing consultant who will work together with PSI and other implementing agencies to provide comparable estimates of the costs of future nation-wide interventions. The indicators to be measured include:

A. Implementation cost

B. The cost-effectiveness in terms of the incremental cost per additional child receiving prompt AL treatment

For the cost-effectiveness indicator, the ‘effectiveness’ measure will be obtained from the household survey. Potential differences in effectiveness between pilot and nation-wide implementation will be assessed using sensitivity analysis.

Feasibility of the Pilot in the Current Political Situation

In view of the current post election violence, certain measures will be undertaken to ensure the safety of the research study team:

- The security and acceptability of the survey work will be discussed at length with local district ministry staff, police, community elders and KEMRI management before travelling to the field.
- Recruitment of local staff will be sensitive to the ethnic tensions within the selected communities that will be surveyed.

ii) Provisions for data verification, and validation in the field

Field workers selected to administer the questionnaires will be chosen according to their level of field experience. They will be fluent in the local dialects spoken in the chosen districts. They will translate the tools into the local language then back translate them to ensure the questions have been translated correctly. Field workers will also undergo two days of training before baseline and intervention surveys to educate them on the purpose of the study, how to fill in the questionnaires and the quality of work expected from them.

As previously mentioned, the data collection tools (both qualitative and quantitative) have drawn on previous similar surveys [60, 62, 71], with results which have been published in peer reviewed journals. They have been adapted to suit the purposes of this study and will all be piloted prior to the surveys and further adjusted, if necessary. Data collected from the different tools can be compared to validate responses from different sources.

During the surveys, questionnaires will be reviewed at the end of each day by the team leaders to ensure all entries are completed appropriately. Any tools with incomplete or questionable entries will be sent back to be re-filled the next day. In 10% of the interviews in the retail census, the household survey and provider survey, field workers will be accompanied by the team leader to monitor their performance and see where any improvements can be made. Also, a random sample of 5% of interviewees in each area will be re-interviewed by team leaders (back-checking) to ensure concordance in responses. The focus group discussions will take place with the principal investigator (BB Kangwana) and an interpreter.

9) Data Management

9a) Data Storage

All data collected from the surveys will be cleaned before and after data entry and will be stored on PCs and USB drives at the KWTRP unit in Nairobi. All data on computers will be secured through the use of passwords. Tapes from the qualitative analyses, field notes and hard copies of the questionnaires will be stored in locked filing cabinets on site. All data entered into software will only have individual identification numbers. Access to any of these materials will be limited to investigators and when necessary clerks for data entry purposes.

9b) Data Management/ Data Analysis

Quantitative Data: Data collected from the retail census, household survey, provider and mystery shopper tools will be entered into individual customized data entry screens on MySQL (Version 5.1, California). These screens will be designed to be as similar as possible to the hard copies with consistency checks and built in ranges. Data will be double entered into the system by two different clerks. Microsoft Excel 2007 and STATA (version 9 StataCorp, College Station, Texas) will be used to analyse these data. To account for the different sampling weights of the EAs in the districts selected, estimates of proportions and means will be calculated using STATA's '*svy*' commands. A wealth indicator will be calculated using PCA of household assets. From this, quintile distribution will be derived [67].

Qualitative Data: Data collected from the focus group discussions will be entered and transcribed in NVivo 7.0 software (QSR International). This software will allow data to be coded systematically into themes and sub-themes that can be evaluated to highlight the perspectives of households and retailers on the retail intervention package.

10) Time Frame

	2008					2009						2010	
	M-A	M-J	J-A	S-O	N-D	J-F	M-A	M-J	J-A	S-O	N-D	J-F	M-A
KWTRP tool pilots													
Retail census													

Baseline Data Collection													
Data entry, coding and cleaning													
PSI AL intervention[+]													
Retail census updated													
Post Intervention data collection													
Data entry, coding and cleaning													
Focus Group Discussions													
Analysis of quantitative and qualitative data													
Report and dissemination meetings													

[+] See *appendix 5* for a detailed timeline of the PSI, AL intervention.

11) Ethical Consideration

This pilot was designed to provide the DOMC and MOH with the evidence they require to inform the decision on whether AL should be rolled out into the private retail sector, and how. The DOMC participated and approved in designing a list of key indicators which they feel are important for their decision making process. The key indicators also incorporate DOMC targets. All information collected from this study will be fed back to the DOMC to inform policy.

Community Engagement: Prior to baseline and post-intervention surveys, letters will be sent to the relevant DHMTs and District Officers (DOs). These letters will explain the study and its purpose. On arrival, the field team leader will meet with both parties, present a copy of the letter and verbally discuss the planned field work. During these discussions concerns or queries about the survey may be voiced. Once approval has been gained a representative from the DHMT will escort the team leader to the relevant village elders, chiefs and/ or local leaders. They will be given a copy of the letter given to the DHMTs together with a verbal explanation. Queries and concerns will be addressed during this time and verbal approval will be sought to go ahead with the survey. The DHMTs, DOs and community leaders will have access to the team throughout the survey so any concerns that arise during the survey can be addressed to the field team leader.

Individual consent: During the surveys, consent will be obtained from the necessary individuals. Informed sheets and consent forms will be translated into the local dialect. A copy of the information sheet will be left for the household heads and shopkeepers. The information sheet will include an introduction, the purpose of the study, how questions will be administered, the risks and benefits to those who participate, that the data collected will be confidential and that participation is purely on a voluntary basis. Written or verbal consent will be obtained prior to baseline and intervention surveys. All verbal consent will be witnessed by a study team member. For the household survey written consent will be gained from the household head or his/ her representative and verbal consent from all other interviewees. For the retail census and

provider surveys verbal consent will be obtained from the interviewees; for the focus group discussions, verbal consent will be obtained from the caregivers and from the shopkeepers. Verbal consent for the mystery shopper survey will be obtained from the shopkeepers during the retail census survey. In all surveys interviewing shopkeepers, verbal as oppose to written consent will be obtained since previous experience shows that shopkeepers tend to be very cautious of inspectors and suspicious of having to sign documents that may be used to identify them or their shop.

Participants will be able to drop out at anytime within or between surveys and do not have to respond to questions they do not wish to. However, reasons for not participating or not answering questions will be obtained as this may be due to aspects of the surveys or intervention that are not acceptable to the community and that will therefore need to be acknowledged and possibly altered. Great efforts will be made to ensure that participant's confidentiality will be maintained at all times. This includes restricting access of all data to the investigators and data entry clerks when need be, destroying of tapes from FGDs after the research is completed, as well as using individual identification numbers on software programs for data analysis.

Training for those involved in administering consent: All fieldworkers will undergo training prior to both studies. Training will educate fieldworkers on the purpose of the study, the importance of consent and how to administer both the consent forms and questionnaires. While in the field, fieldworkers will have daily contact where possible through the use of a mobile phone with the team leaders and KWTRP headquarters in case of any queries.

One potential concern is that AL will be temporarily de-regulated in the study location from a POM to an over the counter medicine (OTC). AL has been used in public facilities for one year, however very little evidence from wide scale distribution is available on the range and impact of adverse effects of AL as an OTC medicine. The use of AL in this study will only be allowed after approval from the PPB. Retailers will be trained on when to refer patients to the public health sector; this information will also be printed on the pre-packaged AL leaflets.

The control areas will not have access to AL as an OTC medicine however, the government will continue providing it free of charge within the public sector in all districts. Therefore the existing access to AL in the control group will not be adversely affected.

During this pilot, retail sector AL will be heavily subsidized within the intervention group which could raise questions on sustainability. If, from all the available evidence the DOMC are willing to distribute AL within the community then there is a high possibility that funds to subsidise this medication at this level/ scale will become available through the implementation of Global Subsidy Funding for ACTs [72].

Feedback of information: Please refer to item 12: expected application of results.

11 b) Animal Subjects: Not applicable

12) Expected Application of Results

The results from this study will be fed back to the DOMC, DHMTs in the study sites, and other interested stakeholders. This will occur together with other studies taking place within and beyond Kenya on retail sector ACT delivery and other strategies to improve access. This pilot will form part of the evidence required by the DOMC to inform the decision on whether distributing AL through the private retail sector will increase prompt access of effective anti-malarial treatment to those who may benefit from it. The evidence may also be useful to other countries thinking of rolling out similar interventions and to national and international organizations who may be willing to fund such interventions at a national level.

The findings will be communicated to relevant and interested stakeholders through written full reports, summary briefing notes, group discussions and one on one meetings, published papers and presentations at international conferences.

13) References

[1] Guerra C, Snow R, Hay S. Mapping the global extent of malaria in 2005. Trends in parasitology. 2006; 22(8):353-8.

[2] Snow R, Guerra C, Noor A, Myint H, Hay S. The global distribution of clinical episodes of *Plasmodium falciparum* malaria. Nature. 2005;434:214-7.

[3] Snow R, Craig M, Deichmann U, Marsh K. Estimating mortality, morbidity and disability due to malaria among Africa's non-pregnant population. Bulletin of the World Health Organization. 1999;77(8):624-40.

[4] Snow R, Gouws E, Omumbo J, Rapuoda B, Craig M, Tanser F, *et al.*, Models to predict the intensity of Plasmodium falciparum transmission: applications to the burden of disease in Kenya. Transactions of the Royal Society of Tropical Medicine and Hygiene. 1998;92(6):601-6.

[5] Fegan G, Noor A, Akhwale W, Cousens S, Snow S. Effect of expanded insecticide-treated bednet coverage on child survival in rural Kenya: a longitudinal study. Lancet. 2007;370:1035-1039.

[6] Roll Back Malaria (RBM), World Health Organisation (WHO). The roll back malaria strategy for improving access to treatment through home management of malaria; 2005.

[7] World Health Organisation (WHO). World Malaria Report; 2005.

[8] Nchinda T. Malaria: a re-emerging disease in Africa. Emerging Infectious Diseases. 1998;4(3):398-403.

[9] Institute of Medicine of the National Academies. Saving lives, buying time. Washington DC, USA: Institute of Medicine of the National Academies 2004.

[10] International Artemisinin Study Group. Artesunate combinations for malaria. Lancet. 2004; 363 9-17.

[11] de Vries P, Dien T. Clinical pharmacology and therapeutic potential of artemisinin and its derivatives in the treatment of malaria. Drugs. 1996;52(6):818-36.

[12] White N. Antimalarial drug resistance and combination chemotherapy. Philosophical Transactions of the Royal Society of London. 1999;354:739-49.

[13] Roll Back Malaria (RBM), World Health Organisation (WHO). Facts on ACTs (Artemisinin-based Combination Therapies). 2006 [cited; Available from: http://www.rbm.who.int/cmc_upload/0/000/015/364/RBMInfosheet_9.htm: Date accessed:15/11/2007.

[14] Division of Malaria Control (DOMC). Transitional plan for implantation of Artemisinin-based combination therapy (ACT) malaria treatment policy in Kenya. Royal Pharmaceutical Management Plus.

[15] Amin A, Zurovac D, Kangwana B, Greenfield J, Otieno D, Akhwale W, *et al*, The challenges of changing national malaria drug policy to artemisinin-based combination in Kenya. Malaria Journal. 2007;6(72).

[16] Zurovac D, Njogu J, Akhwale W, Hamer D, Snow R. Translation of artemether-lumefantrine treatment policy into paediatric clinical practice: an early experience from Kenya. 2007. Manuscript submitted for publication.

[17] Zurovac D, Ndhlovu M, Rowe A, Hamer D, Thea D , Snow R. Treatment of paediatric malaria during a period of drug transition to artemether-lumefantrine in Zambia: a cross sectional study. British Medical Journal. 2005;331.

[18] Rowe A, Hamel M, Flanders W, Doutizanga R, Ndoyo J, Deming M. Predictors of correct treatment of children with fever seen at outpatient health facilities in the Central African Republic. American Journal of Epidemiology. 2000;151:1029-35.

[19] Rowe A, Onikpo F, Lama M, Deming M. Risk and protective factors for two types of error in the treatment of children with fever at outpatient health facilities in Benin. International Journal of Epidemiology. 2003;32:296-303.

[20] Osterholt D, Rowe A, Hamel M, Flanders W, Mkandala C, Chizani N, *et al.*, Predictors of two types of treatment errors for children with malaria seen as outpatients in Blantyre District, Malawi. Tropical Medicine and International Health. 2006; 11:1147-56.

[21] Zurovac D, Rowe A. Quality of treatment for febrile illness among children at outpatient facilities in sub-Saharan Africa. Annals of Tropical Medicine and Parasitology. 2006;100:283-96.

[22] Naimoli J, Rowe A, Lyaghfouri A, Larbi R, Lamrani A. Effect of the Integrated Management of Childhood Illness strategy on health care quality in Morocco. International Journal for Quality in Health Care. 2006;18:134-44.

[23] Wasunna B, Zurovac D, Goodman C, Snow R. Why don't health workers prescribe ACT? A qualitative study of factors affecting the prescription of Artemether-lumefantrine. 2007. Manuscript submitted for publication.

[24] Andrews M. Population Service International 2007. Personal Communication.

[25] Amin A, Marsh V, Noor A, Ochola S, Snow R. The use of formal and informal curative services in the management of paediatric fevers in four districts in Kenya. Tropical Medicine and International Health. 2003;8(12):1143-52.

[26] Abuya T, Mutemi W, Karisa B, Ochola S, Fegan G, Marsh V. Use of over the counter malaria medicines in children and adults in three districts in Kenya: implications for private medicine retailer interventions. Malaria Journal. 2007;10(6).

[27] Gitonga C, Amin A, Ajanga A, Kangwana B, Noor A, Snow R. The use of artemether-lumefantrine by febrile children following national implementation of revised drug policy in Kenya. 2007. Manuscript submitted for publication.

[28] Guyatt H, Snow R. The management of fevers in Kenyan children and adults in an area of seasonal malaria transmission. Transactions of the Royal Society of Tropical Medicine and Hygiene. 2004;98:111-5.

[29] Makinen M, Rauch M, Almagambetova N, Bitran R, Gilson L *et al.*, Inequalities in health care use and expenditures: empirical data from eight developing countries and countries in transition. Bulletin of the World Health Organisation. 2000;78(1):55-65.

[30] Hotchkiss D, Rous J, Karmacharya K, Sangraula P. Household health expenditures in Nepal: implications for health care financing reform. Health Policy and Planning. 1998;13(4):371-83.

[31] Okonkwo P, Akpala C, Okafor H, Mbah A, Nwaiwu O. Compliance to correct dose of chloroquine in uncomplicated malaria correlates with improvement in condition of rural Nigerian children. Transactions of the Royal Society of Tropical Medicine and Hygiene. 2001;95(3):320-4.

[32] Patouillard E, Goodman C, Hanson K, Mills A. Can working with the private for profit sector improve utilisation of quality health services by the poor? A systematic review of the literature. International Journal for Equity in Health. 2007;6:17.

[33] Tavrow P, Shabahang J, Makama S. Vendor-to-vendor education to improve malaria treatment by private drug outlets in Bungoma District, Kenya. 2003. Manuscript submitted for publication.

[34] Kolaczinski J, Webster J. Malaria control in complex emergencies: the example of East Timor. Tropical Medicine and International Health. 2003;8(1):48-55.

[35] Denis M. Improving compliance with quinine + tetracycline for treatment of malaria: evaluation of health education interventions in Cambodian villages. Bulletin of the World Health Organisation. 1998;76(suppl 1):43-9.

[36] Ruebush T, Kern M, Campbell C, Oloo A. Self treatment of malaria in a rural area of western Kenya. Bulletin of the World Health Organisation. 1995;73(2):229-36.

[37] Foster S. Treatment of malaria outside the formal health service. Journal of Tropical Medicine and Hygiene. 1995;98(1):29-34.

[38] Kidane G, Morrow R. Teaching mothers to provide home treatment of malaria in Tigray, Ethiopia: a randomised trial. Lancet. 2000;356:550-5.

[39] Kofoed P, Lopez F, Aaby P, Hedegaard K, Rombo L. Can mothers be trusted to give malaria treatment to their children at home? Acta Tropica. 2003;86(1):67-70.

[40] Yip W, Berman P. Targeted health insurance in a low income country and its impact on access and equity in access Health Economics. 2001;10(3):207-20.

[41] Alilio M, Kamugisha M, Msuya F MJ, Salum F, Njunwa KJ. Availability and utilization of anti-malarial drugs at community level in Same District North Eastern Tanzania. Malaria and Infectious Diseases in Africa. 1997;6.

[42] Baume C, Helitzer D, Kachur S. Patterns of care for childhood malaria in Zambia. Social Science and Medicine. 2000;51(10):1491-503.

[43] Oshiname F, Brieger W. Primary care training for patent medicine vendors in rural Nigeria. Social Science and Medicine. 1992;35(12):1477-84.

[44] Marsh V, Mutemi W, Muturi J, Haaland A, Watkins W, Otieno G, *et al.*, Changing home treatment of childhood fevers by training shop keepers in rural Kenya. Tropical Medicine and International Health. 1999;4(5):383-9.

[45] Ndomondo-Sigonda M, Kowero O, Alphonse E, Hebron Y Kihinga, C Mbwasi R, Shirima R, Taylor M, Heltzer N, Clark M. Accredited Drug Dispensing Outlets: A Novel Public-Private Partnership. Dar es Salaam, Tanzania: Tanzania Food and Drugs Authority, Tanzania, Healthscope, and MSH/SEAM. SEAM (Strategies for Enhancing Access to Medicines) Conference; 2003.

[46] Ndyomugyenyi R, Neema S, Magnussen P. The use of formal and informal services for antenatal care and malaria treatment in rural Uganda. Health Policy and Planning. 1998;13:94-102.

[47] Rozendaal J. Fake antimalaria drugs in Cambodia. Lancet. 2001;357:890.

[48] Pagnoni F, Convelbo N, Tiendrebeogo J, Cousens S, Esposito F. A community-based program to provide prompt and adequate treatment of presumptive malaria in children. Transactions of the Royal Society of Tropical Medicine and Hygiene. 1997;91(5):512-7.

[49] Ansha E, Gyapong J, Agyepong I, Evans D. Improving adherence to malaria treatment for children: The use of pre-packed chloroquine tablets vs. chloroquine syrup. Tropical Medicine and International Health. 2001;6(7):496-504.

[50] Ombogo J, GSMF Enterprises Ltd, Management Sciences for Health. The Child and Family Wellness Shops Story: Improving access to live-saving medicines through micro-franchising. . SEAM (Strategies for Enhancing Access to Medicines) Conference; 2005; Accra, Ghana: 2005.

[51] Magesa S, Lengeler C, deSavigny D, Miller J, Njau R, Kramer K, *et al.*, Creating an enabling environment for taking insecticide treated nets to national scale, the Tanzanian experience. Malaria Journal. 2005;4:34.

[52] Hopkins H, Talisuna A, Whitty J, Staedke S. Impact of home-based management of malaria on health outcomes in Africa: a systematic review of the evidence. Malaria Journal. 2007;6:134.

[53] Goodman C, Brieger W, Unwin A, Mills A, Meek S, Greer G. Medicine sellers and malaria treatment in sub-saharan Africa: what do they do and how can their practice be improved? The American Journal of Tropical Medicine and Hygiene. 2007; 77(suppl 6):203-218.

[54] Goodman C, Mutemi W, Baya E, Willetts A, Marsh V. The cost effectiveness of improving malaria home management: shopkeeper training in rural Kenya. Health Policy and Planning 2006;21:275-88

[55] Marsh V, Mutemi W, Muturi J, Haaland A, Watkins W, Otieno G, et al. Changing home treatment of childhood fevers by training shop keepers in rural Kenya. Tropical Medicine and International Health. 1999;4(5):383-9.

[56] Marsh VM, Mutemi WM, Willets A, Bayah K, Were S, Ross A, *et al.*, Improving malaria home treatment by training drug retailers in rural Kenya. Tropical Medicine and International Health. 2004;9:451-60.

[57] World Health Organisation (WHO). Guidelines for the treatment of malaria; 2006. Report No.: ISBN 978 92 4 154694 2.

[58] McCombie S. Self-treatment for malaria: the evidence and methodological issues. Health Policy and Planning. 2002;17(4):333-44.

[59] Abuya T. Kemri Wellcome Trust Reseach Programme. Nairobi 2007. Personal Communication

[60] Amin A. Range, quality, and costs of antimalarial drugs available in the retail sector in Kenya: Open University, United Kingdom; 2005.

[61] Goodman C, Kachur SP, Abdulla S, Mwageni E, Nyoni J, Schellenberg JA, et al. Retail supply of malaria-related drugs in rural Tanzania: risks and opportunities. Tropical Medicine and International Health. 2004;9(6):655-63.

[62] The CORE Group MIHV. Improving malaria case management in Ugandan communities: Lessons from the field. Washington DC; 2004.

[63] Noor A. Developing spatial models of health service access and utilization to define health equity in Kenya: Open University, United Kingdom; 2005.

[64] McCombie S. Self-treatment for malaria: the evidence and methodological issues. Health Policy and Planning. 2002;17(4):333-44.

[65] Patouillard E, Goodman C, Hanson K, Mills A. Can working with the private for profit sector improve utilisation of quality health services by the poor? A systematic review of the literature. International Journal for Equity in Health. 2007;6:17.

[66] Reidpath D, Allotey P. Measuring global health inequity. International Journal for Equity in Health. 2007;6:16.

[67] Noor A, Omumbo J, Amin A, Zurovac D, Snow RW. Wealth, mother's education and physical access as determinants of retail sector net use in rural Kenya. Malaria Journal 2006 26(5):5.

[68] Marsh V, Mutemi W, Willets A, Bayah K, Were S, Ross A, *et al.*, Improving malaria home treatment by training drug retailers in rural Kenya. Tropical Medicine and International Health. 2004;9:451-60.

[69] Madden J, Quick J, Ross-Degnan D, Kafle K. Undercover careseekers: simulated clients in the study of health provider behavior in developing countries. Social Science and Medicine. 1997;45(10):1465-82.

[70] Chalker J, Falkenberg T, Tomson G. STD management by private pharmacies in Hanoi: practice and knowledge of drug sellers. Sexually Transmitted Infections. 2000 76:299-302.

[71] Goodman C. An economic analysis of the retail market for fever and malaria treatment in rural Tanzania. London: London School of Hygiene and Tropical Medicine, University of London; 2004.

[72] Global ACT Task Force. Increasing access to malaria medicines. Draft Technical Proposal for a Global ACT Subsidy 2007.

14) Budget

Item	Amount(US$)	Amount (Kshs)
a) Personnel salaries and benefits	151,054	9,969,564
b) Patient Costs	NA	NA
c) Equipment	NA	NA
d) Supplies	3,840	253,440
e) Travel and accommodation	14,800	976,800
f) Transportation	NA	NA
g) Operating expenses	196,305	12,956,130
h) Animals	NA	NA
i) Consultancy fees	NA	NA
j) Contingency funds	NA	NA
k) Institutional administrative overheads	45,784	3,021,744
Total	411,783	27,177,678

15) Justification of Budget

This budget only covers the cost for the evaluation of the pilot. All intervention costs will be estimated and finance sort by PSI.

Personal Salaries and Benefits: The principal investigator, (BB Kangwana) and an assistant research officer will be paid for a period of 24 months. The role of the assistant research officer will be to monitor the progress of the surveys in the field. Salaries for the research assistant and assistant research officer have been calculated using the appropriate KWTRP salary scales. Salaries also include 8 months for Catherine Goodman who will provide the majority of supervisory support. She will be paid according to the London School of Hygiene and Tropical Medicine salary scale.

Operating Expenses: This includes all costs required to carry out the field studies. The retail census, household survey, provider survey and mystery shopper survey will be carried out twice over a period of two years, at baseline and post intervention. The focus group discussions will only be carried out post intervention while documentation of context will be collected throughout the study period. The operating costs include travel costs, per diems and salaries of the field workers as per KWTRP guidelines. Training days will be held before each survey, one for the team leaders, one for the field workers on data collection, and one for all staff on the use of GPS units to map households and shops. Salaries and transportation costs will need to be paid to those attending the training days. Once in the field, village elders will be asked to help in locating the households and shops and will be paid 100ksh per day. The salaries and per diems are included for one field worker and a team leader to remain in the field for an extra three days after the surveys are complete to deal with any remaining errors that need to be corrected (call backs). Also included are costs for the production of the questionnaires, costs to pilot the questionnaires, the salaries for a software developer to design the data entry screens and clerks for data entry purposes and the costs to purchase 11 GPS units.

Supplies: This is the estimated cost of all the non-field work supplies required for the study at the KWTRP unit in Nairobi. It includes photocopying, stationary, communication and printing over the two year period. The estimates are based on routine costs charged at the KWTRP.

Travel and Accommodation: During the protocol development and study preparation an investigator will need to meet with a statistician and retail sector consultants in Kilifi. Travel and accommodation costs for these trips have been factored in here. Again, while the surveys are going on, an investigator will need to travel to the field sites for a couple of days to monitor the progress on the ground, check the quality of the data collection and deal with any major issues that may present. All costs for travel and accommodation have been estimated using standard KWTRP mileage and per diem charges.

16) Appendices

16a) Role of Each Investigator

Miss BB Kangwana: PI- Will be responsible for proposal development, supervision of training, data collection, analysis and report preparation and communication.

Dr C Goodman: Will play a supervisory role in designing and co-ordinating proposal development and provide scientific guidance for the study.

Dr G Fegan: Will provide statistical support in study design and analysis.

Dr AM Noor: Will be involved in developing GIS platforms and evaluation of socio-economic status.

Dr AJ Nyandigisi: Program pharmacist, Division of Malaria Control. Will be the principal representative/ liaison person for the DOMC. Will be responsible for coordinating planned HMM pilots for DOMC purposes and, in informing policy discussions on issues raised in the protocol and subsequent publications arising from the protocol.

Dr WS Akhwale: Head of Division of Malaria Control, Ministry of Health; will be responsible for informing policy discussions on issues raised in the protocol and subsequent publications arising from the protocol.

Pharmacy and Poisons Board: Will act as an advisory body on pharmaceutical issues in the study, responsible for informing policy decisions on issues raised in the protocol and subsequent publications arising from the protocol. The PPB will also be responsible for the monitoring and evaluation of ADRs. This includes working with PSI and the DOMC in distribution of referral forms, training of shop keepers and health care workers on how to identify and record ADRs, and collection and analysis of data from referral forms and any other tools used to record ADRs..

Dr Jayesh Pandit: Head of Pharmacovigilance, Pharmacy and Poisons Board, Ministry of Health; will be the principal representative/ liaison for the PPB, co-ordinating communications between the PPB and other participatory bodies.

Prof RW Snow: Responsible for overall scientific guidance for the study, its interpretation and write up.

16b) Attached relevant documents

APPENDIX 1

CVs of non-KEMRI investigators

Name: **Willis Akhwale**

Position: Head, Division of Malaria Control, Ministry of Health, Nairobi, Kenya.

Education:

- University of Nairobi, College of Health Sciences, Bachelor of medicine and surgery (MbChB),1991.
- Aga-Khan, Nairobi, Diploma in Primary Health Care, 1996
- Tokyo Women`s Medical University, Tokyo, Japan, PhD in Tropical Medicine, 2000.

Previous Positions:

1991-1992 Rift Valley Provincial General Hospital, Nakuru, Kenya, internship in medicine, paediatrics, surgery, obstetrics and gynaecology.

1992-1993 Medical officer in the surgery department, Rift Valley Provincial General Hospital Nakuru.

1993-2000 District Medical Officer of Health, Trans Nzoia district.

Publications:

- Fegan GW, Noor AM, **Akhwale WS**, Cousens S & Snow RW (2007). Effect of expanded insecticide-treated bednet coverage on child survival in rural Kenya: a longitudinal study. *Lancet*, 370:1035-9.
- Noor AM, Amin AA, **Akhwale WS** & Snow RW (2007). Increasing coverage and decreasing inequity in insecticide-treated bed net use among rural Kenyan children. *PLoS Medicine*, 4:e255.
- Amin AA, Zurovac D, Kangwana BB, Greenfield J, Otieno DN, **Akhwale WS** & Snow RW (2007). The challenges of changing national malaria drug policy to artemisinin-based combinations in Kenya. *Malaria Journal*, 6:72.
- Zurovac D, Larson BA, **Akhwale WS** & Snow RW (2006).The financial and clinical implications of adult malaria diagnosis using microscopy in Kenya. *Tropical Medicine and International Health*, 11:1185-94.
- **Akhwale WS**, Lum JK, Kaneko A, Eto H, Obonyo C, Bjorkman A & Kobayakawa T. (2004). Anaemia and unstable malaria at different altitudes in Kisii District of the highlands of western Kenya. *Acta Tropica*, 91:167-7

Name: **Andrew J. Nyandigisi**

Position : Program Pharmacist Division of Malaria Control in charge of community access of antimalarials, pharmacovigilance and QA/QC of anti-malarials .

Education

- University of Nairobi, College of Health Sciences, Bachelor of Pharmacy 2004
- Training on overview of Supply Chain Management for Commodity Security by JSI deliver (Johannesburg)
- Training on Minilabs for QC of anti-malarials by USP (Addis Ababa)

Previous positions

- Hospital Pharmacist Kenyatta National Hospital
- Internship in Community pharmacy Lemuma Pharmacy
- Internship in Industrial Pharmacy ELYS chemical industries

APPENDIX 2

Household Survey Sample Size Calculation Using Hayes and Bennett Cluster Sampling Formula IJE 1999 [8]

An alternative way to calculate sample size is by using the Hayes and Bennett Cluster Sampling formula:

$c = 2 + 7.84\ [p1\ (1\text{-}p1)/n + p2\ (1\text{-}p2)/n + k^2(p1^2 + p2^2)]\ /\ (p1 - p2)^2$

(Eq 4 adjusted for matching by adding 2)

Where

c = number of clusters

n = Average expected number of fevers per cluster

k = co-efficient of variation, estimated at 0.25, based on figures used by Hayes and Bennett

- ⇨ n = (20 HS per EA) * (no. of fevers per HS) = 5 (rounded down)
- ⇨ c = 21 EAs in intervention group and 21 in control group
- ⇨ i.e. 2.4 EAs per division.

If increased k to 0.35 would require 23 EAs in each group and therefore 2.6 EAs per sub location.

This implies that the 3 EAs per sub location estimated in approach a) above can be considered adequate.

---

[8] Hayes RJ, Bennett S. Simple sample size calculation for cluster-randomized trials. International Journal of Epidemiology, Vol 28, 319-326.

APPENDIX 3

Sample Size Calculation for ACT Retail Study

**Parameters**		
z_alpha/2	1.96	standard
z_beta	0.84	standard
p_control	0.2	Fever survey June 07 gave 11% but increased to be conservative (proportion with access to AM within 48 hours)
p_intervention	0.4	Assuming a 20% point increase after intervention
No. HS to get one fever	3.4	average across survey data (exc. Makueni)
Design effect	2	estimate
Sub-locations per group	9	study design
k	0.25	estimate used in Hayes & Bennett - based on 2001/2 survey data we had calculated 0.16 so 0.2 could be considered conservative
number of HS sampled per cluster	20	chosen based on being about to find at least 20 HS in most EA as IQR is 23-41
number of fevers per cluster	5	

**Calculation based on individual randomised study plus design effect**		
n	79	fevers in each group
No. HS	269	
No. HS + design effect	538	
No. HS per sub-location	60	
EA sampled per sub-location	3	
EA per intervention group	27	

**Calculation based on cluster randomised study using Hayes & Bennett equation 4, adjusted for matching**	
c, number of clusters	21.00
EA per sub-location	2.4

APPENDIX 4

Key Indicators for Evaluating Interventions to Expand ACT Coverage in Kenya

Background

Kenya has been delivering artemisinin combination therapy (ACT) through the public sector since mid-2006. Early evaluations show this led to an increase in the percentage of children under 5 receiving antimalarials from 14% to 26% within 48 hours, of whom 30% received artemether- lumefantrine (AL) (KEMRI/Wellcome Trust, 2007). However, access remains well below the Roll Back Malaria target of 60%, with the majority of patients still failing to receive prompt effective treatment. The Ministry of Health (MOH) is committed to exploring alternative delivery channels that can complement existing facility-level delivery, and thus improve community access to ACT. These could include delivery through drug retailers, Sustainable Health Enterprise Foundation (SHEF) clinics, Community Owned Resource Persons (CORPs) or other volunteers. Pilots of such interventions have been proposed by a number of groups, and the Pharmacy and Poisons Board (PPB) is expected to approve a special dispensation to allow over the counter (OTC) status for AL for these pilots.

The Division of Malaria Control (DOMC) is keen to ensure that evidence arising from these pilots is of high quality and based on common indicators, to facilitate comparison of results and identification of policy implications. This paper therefore outlines core indicators which should be included in all studies, and identifies optional complementary indicators which may also be included. In addition, each pilot may wish to add their own indicators that address outcomes specific to their interventions or interests.

The indicators have been identified through consideration of DOMC targets, Global Fund (GF) and Roll Back Malaria Monitoring and Evaluation Reference Group (RBM MERG M&E) frameworks, and the draft monitoring and evaluation (M&E) guidelines for the global ACT subsidy.

Guidelines on Study Design

All studies must include either:

- Pre and post intervention data
- Intervention and control groups

It is strongly recommended that both are included (i.e. pre and post for intervention and control groups). However, this may be infeasible for some studies, for example where the intervention has already begun, or where no appropriate control groups exist.

Cluster randomization of outlets to intervention and control groups reduces the potential for bias. However, it may be infeasible for some interventions which need to function at a certain minimum scale, or where “contamination” would be likely between clusters (e.g. residents from control clusters could visit outlets in intervention clusters or receive communication messages targeted at the intervention group). In most cases it is therefore likely that there may be only 1 or 2 control and intervention areas.

Study Indicators

Indicators have been divided into 3 groups:

A. **Indicators to be included in all studies (please refer to back page for summary)**
B. **Optional complementary indicators**
C. **Indicators likely to be beyond the scope of pilot studies**

A. Indicators to be Included in all Studies

A1: Household Survey Indicators

Indicator 1: The proportion of children under 5 years with fever in the past 2 weeks who started treatment with a first line ACT within 24 and 48 hours of fever onset, overall, by socio-economic group (SEG) and treatment source

- This will be the primary indicator for all studies. It is a standard RBM indicator collected as part of the Multiple Indicator Custer Surveys (MICS) and the Demographic and Health Surveys (DHS) and a core GF indicator.
- It requires collection through a household survey, which will represent an additional expense for some pilots. However, it is essential in order to measure the overall impact on ACT coverage from both facility and non-facility sources. This is important as an intervention with high levels of ACT provision through non-facility sources would not be considered a success if it reduced facility provision, and possibly even reduced ACT coverage overall.
- All compulsory household survey indicators focus on children under 5 years as the most biologically vulnerable group. Older groups are also important, but their inclusion would increase the complexity of the household survey. The same indicators for these groups are therefore considered optional.
- 2 weeks is the standard recall period for this indicator.
- We focus on fever (rather than malaria) as the majority of febrile illnesses do not receive parasitological confirmation, and fever is the main symptom used in clinical diagnosis. In addition, for most communities in Africa, fever is the prompt for seeking treatment.
- We have included both 24 and 48 hours (although RBM targets have been specified in terms of 24 hours), as treatment within 48 hours could still be considered prompt. The denominator for 24 (48) hours should be all individuals reporting fever, visited 2 (3) or more days after symptoms began. Surveys must therefore also ask when symptoms began.
- The indicator should be collected by SEG in view of the emphasis placed on equity by both the Kenyan MOH, and RBM. All surveys should therefore include the standard asset indicators from the Kenya (K)DHS (collection of asset indicators is much quicker and more reliable than collection of income or expenditure data). Households should then be allocated to national SEGs on the basis of national KDHS weights. This is preferable to calculating a study specific asset index and SEGs, as, for example, households in socio-economic quintile 3 in one study area could be in quintile 5 in another.
- The indicator should be collected by treatment source (e.g. public, faith-based organisations (FBOs) and commercial facilities, pharmacies, other retail outlets and CORPs) in order to know through which channels the intervention is achieving its goals.
- Study teams should choose a sample size capable of detecting at least a 20 percentage point increase in ACT coverage in the target group (5% significance, 80% power, allowing for clustering if such sampling is used). As a rough guide, assuming an initial proportion of 11% (KEMRI/Wellcome Trust, 2007), with a simple random sample of households this would require a minimum of 65 childhood fevers per group (before and after, or control and intervention). In areas with moderate to high malaria transmission you are likely to need to visit between 2 and 4 households to find one childhood fever, meaning that you should sample at least 260 households in each group. If a cluster design is used it would be conservative to double this requirement to 520 households per group.
- It is recommended that tools such as picture boards are used to facilitate recall of drugs used.

Indicator 2: The proportion of children under 5 years with fever taking a first line ACT who adhered to the treatment dose, by source

- Adherence is important in both treatment efficacy and reducing the risk of the development of resistance.
- As ACT is a 3 day course, the denominator should be individuals interviewed 3 or more days after ACT treatment began.
- The indicator should be measured by source to indicate any variations in adherence across treatment types.
- Adherence will be defined as taking the quantity of drug specified in MOH guidelines by age group over 3 days (i.e. excludes both under and over dosing).
- Timing of doses within the 3 days will not be considered due to problems of precise time recall.

Indicator 3: The proportion of children under 5 years with fever who took an anti-malarial monotherapy in the past 2 weeks

- This assesses whether the intervention has succeeded in crowding monotherapies from the market, which are undesirable because they create competition that may decrease the demand for more effective combination therapies such as ACTs. The availability of artemisinin monotherapies increases the likelihood of the development of resistance to artemisinin, thus reducing the useful therapeutic life (UTL) of the ACTs.

Indicator 4: The proportion of children under 5 years with fever in the past 2 weeks who sought treatment by source (e.g. from public, mission & commercial health facilities, pharmacies, other retail outlets, CORPs, traditional healers and other sources)

- This indicator will allow assessment of any changes in treatment seeking patterns as a result of the intervention, for example whether there is a shift away from facilities, or from shops to CORPs, or an overall increase in the proportion seeking any care.
- The indicator covers any use of each outlet type, irrespective of the order in which they were used. One child may therefore use more than one source.

A2: Provider Survey Indicators

Indicator 5: The median price charged by non-facility outlets for a first line ACT by age band

- The price charged is important in order to assess affordability and whether subsidies are being passed onto final users i.e. are providers adhering to recommended retail prices or to free provision depending on the intervention design.
- We propose collecting drug price data from the provider survey rather than the household survey because of the difficulties of recall of costs in household surveys, the problems of separating the cost of a single drug from other payments, and problems of standardisation due to variation in patient age and dose obtained. It is possible that providers may not admit diverging from recommended prices under direct questioning, so an optional alternative is to validate price data through the patient- provider encounter indicators – see below.
- The median rather than the mean is generally used for cost and price data as the data tend to be skewed.

Indicator 6: The proportion of non-facility outlets with no expired first line ACT available in stock
Indicator 7: The proportion of non-facility outlets reporting stock outs of the first line ACT within the past 2 weeks

- A stock out is regarded as any period of time the facility does not contain stock. Restricting stock outs to a minimum period of time may complicate data collection in addition, an efficient supply chain should ensure that drugs are always available for customers to purchase.

Indicator 8: The proportion of non-facility outlets storing first line ACT appropriately

- Storage conditions include: (1) Off floor, (2) Out of direct sunlight, (3) Dry area, (4) Away from foodstuff, (5) All conditions met. "Appropriate storage" is defined as item (5) i.e. all storage conditions (1-4) have been met.

Indicator 9: The proportion of non-facility outlets that have copies of the materials/ job aids required by the intervention (e.g. leaflets, posters, guidelines)

A3: Intervention Cost Indicators

Ideally one would want cost data from all pilots in order to compare:
Indicator 10: Implementation cost per intervention area

- The cost of the intervention will provide information on the size of budget required to roll out a similar intervention either at a regional or national level.
- Costs should take into account all items that were paid for during the planning and rollout of the intervention. These include for example, costs of purchasing the anti-malarial drugs, costs of transport of staff and goods to and from intervention sites, salaries paid to all staff who played a part in planning and implementation of the intervention, and costs incurred from the development of information, education and communication materials.

Indicator 11: The cost-effectiveness in terms of the incremental cost per additional child receiving prompt ACT treatment
However, there are a number of challenges:

- Pilots tend to operate on a small scale and therefore costs may not be representative of larger scale operations, when costs per capita may fall significantly.
- Considerable effort is required to ensure that cost data collected are comparable.
- Cost data collection and analysis requires specific skills that may not be available to all partners.

A costing consultant will therefore be hired by the MOH to work with each team to estimate costs for scaled up operation of each pilot. This will facilitate the use of standardised methods and unit costs where appropriate, and avoid basing policy decisions on unrepresentative costs from differing small scale operations. It is therefore compulsory for all pilots to keep records of resource use, and to collaborate with the MOH in any costing analysis required.

A4: Pharmacovigilance Indicators

Pilots will be required to collaborate with regulatory authorities (PPB, MOH) to ensure that any pharmacovigilance requirements are met within their intervention design.

B. Optional complementary indicators

B1: Household Survey Indicators

- *Indicators 1-4 could be adapted to consider Individuals of 5 years and over*
- *Indicator 1 could be adapted to consider all antimalarials, or all "effective" antimalarials*
- *The proportion of non-target household members receiving intervention ACT (e.g. adults receiving paediatric ACT if ACT is targeted at children only; pregnant women receiving ACT)*
- *Household cost of fever episode (for all completed episodes)*
- *The proportion of households within 30 minutes to 1 hour travel time from a first line ACT source*
- *The proportion of caregivers with knowledge of malaria symptoms, danger signs, ACT and correct ACT dose for 2 year old*

B2: Provider Survey Indicators

- *Total volume of ACT distributed per capita in public and private sectors*
- *The proportion of sub-locations with at least one ACT source*
- *Availability of public sector ACT in inappropriate outlets*
- *Availability of private sector intervention ACT in inappropriate outlets*
- *The proportion of non-facility outlets with first line ACT stock records that correspond with physical counts*

The above indicators require a detailed understanding of a variety of factors for all service providers in the locality including the numbers, nature and antimalarial stocks. This is unlikely to be feasible for all studies.

B3: Patient- Provider Encounters Indicators

In addition to the household and provider surveys, it is necessary to include some evaluation of patient-provider interaction to give better information on actual (rather than self-reported) provider behaviour. Patient-provider interaction can be assessed by a number of methods including the following:

- Direct observation requires data collectors to directly observe the behaviour of the provider for the purpose of describing over the counter prescribing and dispensing practices.
- Mystery shopper study, where data collectors pose as ordinary customers. It provides similar information to direct observation, except the observer does not have to stay at the site for a substantial period of time; the potential bias from observation is eliminated and the scenarios assessed can be standardised between outlets. This technique does however raise some ethical concerns because informed consent is not obtained from the medicine seller before the study is conducted.
- Exit interviews provide information to determine how well each patient/caregiver understood the instructions given by the provider and also can be used as a record of reported patient provider interactions.
- Vignette surveys. These are short hypothetical scenarios described to the interviewee with the intention of eliciting a response from them. For example 'what would you do if a care giver presented with a 2 year old child with fever?'. The response is used to portray perceptions, opinions, beliefs and attitudes of the interviewee. The responses however do not provide any information on the actions of the care giver if presented with a similar real life situation as beliefs do not always translate into action.

- Management information systems involve using retrospective data collected by the non-facility outlet to determine the prescribing and dispensing practices of the provider

Data will not be strictly comparable between the above methods. However, no single method is possible in all studies for practical reasons. For example, it would not be efficient to do an exit interview where there are only a few customers per day; neither is it possible to use mystery shoppers at a clinic. Each study should therefore include the method most suitable to them.

Indicators for the patient provider encounters include:

- *The proportion of non-facility outlet staff that dispense an appropriate first line ACT to patients presenting with fever*
- *The proportion of non-facility outlet staff that dispense the correct dose of first line ACT to patients presenting with fever*
- *The proportion of non-facility outlets that provide appropriate information to patients/ caregivers on how to give/ take the first line ACT*
- *The proportion of encounters where non-facility outlet staff asked one or more clinical questions to determine severity of malaria*
- *The proportion of non facility outlet staff who told patients/ caregivers about any signs of progressive illness and recommended a referral visit to a doctor or clinic if the signs appear*
- *The median price charged for an ACT dose by age group*

B4: Qualitative Data Indicators

It is also recommended that studies include qualitative data collection activities such as focus group discussions and in-depth interviews with household members, providers and others involved in the distribution chain. These will complement, validate and help interpret the quantitative indicators, providing a richer understanding of the reasons behind achievements and constraints in implementation. Qualitative data may also be used to inform the design of quantitative instruments.

B5: Diagnostic Indicators

Diagnosis can be either clinical (presumptive) or parasitologically confirmed (microscopy or rapid diagnostic tests). It is likely that a mix of diagnostic approaches will be used across the studies. Where parasitologically confirmed diagnosis is used in an intervention, the following indicators should be evaluated:

- *The proportion of non-facility outlets staff able to confirm a malaria diagnosis according to national guidelines using the approved diagnostic*
- *The proportion of non-facility outlet patients who undergo a malaria diagnostic test*
- *The sensitivity and specificity of a diagnostic test*. Diagnostics should be of high sensitivity since false negatives may result in failure to treat, and of high specificity to avoid over diagnosis and subsequent over treatment.
- *The proportion of non facility outlets reporting stock outs of the approved diagnostic or components required for its proper functioning, within the past 2 weeks*

C. Indicators Likely to be Beyond the Scope of Pilot Studies

C1: Morbidity and Mortality Indicators

Final health outcome measures such as number of severe malaria cases and number of malaria fatalities would clearly be desirable. However, they are difficult to obtain for 2 reasons:

- They are relatively rare events and therefore require very large sample sizes;
- Many severe cases and deaths are not seen at health facilities and therefore are difficult to identify; over 60% of deaths in Kenya and in the sub-region occur at home.

It is therefore not expected that most studies will include these indicators. Exceptions may be large scale studies which could consider facility reports of severe cases, or studies taking place within demographic surveillance areas where community-based malaria mortality rates can be assessed based on verbal autopsy.

C2: Drug Resistance Indicators

Pilots are unlikely to be able to measure the impact of their intervention on drug resistance directly through treatment failure rates or genetic resistance markers. Measuring such outcomes would require large sample sizes, long time frames, and considerably increase evaluation costs in terms of collection of blood samples and laboratory analysis.

C3: Drug Quality Indicators

All pilots will use ACT supplies from quality certified sources and will therefore not be at risk of poor quality from sub-standard or fraudulent manufacturing. However, it is possible that the storage and handling of ACT in the supply chain could negatively affect quality, and these intermediate outcomes are therefore included under the provider survey. Again, additional laboratory tests would be required to assess the impact of storage and handling on actual quality.

Summary of Compulsory Indicators

No.	Indicator	Source
**1**	The proportion of children under 5 years with fever in the past 2 weeks who started treatment with a first line ACT within 24 and 48 hours of fever onset, overall, by socio-economic group (SEG) and treatment source	Household Survey
**2**	The proportion of children under 5 years with fever taking a first line ACT who adhered to the treatment dose, by source	Household Survey
**3**	The proportion of children under 5 with fever who took an anti-malarial monotherapy in the past 2 weeks	Household Survey
**4**	The proportion of children under 5 years with fever in the past 2 weeks who sought treatment by source (e.g. public, mission & commercial health facilities, pharmacies, other retail outlets, CORPs, traditional healers and other sources)	Household Survey

**5**	The median price charged by non-facility outlets for a first line ACT by age band	Provider Survey
**6**	The proportion of non-facility outlets with no expired first line ACT available in stock	Provider Survey
**7**	The proportion of non-facility outlets reporting stock outs of the first line ACT within the past 2 weeks	Provider Survey
**8**	The proportion of non-facility outlets storing first line ACT appropriately	Provider Survey
**9**	The proportion of non-facility outlets that have copies of the materials/ job aids required by the intervention (e.g. leaflets, posters, guidelines)	Provider Survey
**10**	Implementation cost per intervention area	Costing Study
**11**	The cost-effectiveness in terms of the incremental cost per additional child receiving prompt ACT treatment	Costing Study (effectiveness from household survey)
**12**	To be confirmed by relevant regulatory authorities	Pharmacovigilance

APPENDIX 5:
PSI Intervention Timelines

	2008												2009												2010
**Activity**	Jan	Feb	Mar	Apr	May	Jun	Jul	Aug	Sep	Oct	Nov	Dec	Jan	Feb	Mar	Apr	May	Jun	Jul	Aug	Sep	Oct	Nov	Dec	Jan
**Administrative Issues**																									
Proposal ethical approval		■	■	■																					
OTC Status Advocacy	■	■																							
**Product**																									
Concept Design		■	■																						
Pre-test				■																					
Final design				■																					
DOMC/PPB Approval				■																					
Product Arrival in country						■																			
Production (overpackaging)							■																		
**Training**																									
Adaptation of diagnostic algorithm		■	■																						
Develop Shopkeeper training manual			■	■																					
Outlet selection & Recruitment							■																		
Train shopkeepers									■	■															
Train IPC teams							■	■																	
**Communications**																									
Develop & produce communication materials				■	■	■																			
Develop & produce POS materials				■	■	■																			
Disburse BTL promotional material								■																	
**Launch**																									
Sell Into Trade											■	■	■	■	■	■	■	■	■	■	■	■	■	■	■
Franchising/Merchandising outlets											■	■	■	■	■	■	■	■	■	■	■	■	■	■	■
Demand creation activities (IPC)											■	■	■	■	■	■	■	■	■	■	■	■	■	■	■

APPENDIX 6

Abbreviations/ Acronyms

**ACT**	Artemisinin Combination Therapy
**ADR**	Adverse Drug Reactions
**AIDS**	Acquired Immuno-Deficiency Syndrome
**AL**	Artemether- Lumefantrine
**AQ**	Amodiaquine
**CORPs**	Community Owned Resource Persons
**DHMT**	District Health Management Team
**DHS**	Demographic and Health Survey
**DO**	District Officer
**DOCH**	Division of Child Health
**DOMC**	Division of Malaria Control
**DPTWG**	Drug Policy Technical Working Group
**EA**	Enumeration Area
**EST**	Estimated
**FDG**	Focus Group Discussions
**GDP**	Gross Domestic Product
**GF**	Global Fund
**GPS**	Global Positioning System
**HH**	Households
**HIV**	Human Immuno-deficiency Virus
**HMM**	Home Management of Malaria
**HS**	Homestead
**IEC**	Information, Education and Communication
**IMCI**	Integrated Management of Childhood illness
**ITN**	Insecticide Treated Net
**Kshs**	Kenya Shillings
**KWTRP**	Kemri Wellcome Trust Research Programme
**MOH**	Ministry of Health
**NGO**	Non Governmental Organization
**NO**	Number
**OTC**	Over the Counter
**PCA**	Principal Component Analysis
**PMI**	President's Malaria Initiative

**POM**	Prescription Only Medicine
**POP**	Population
**PPB**	Pharmacy and Poisons Board
**PSI**	Population Services International
**RBM**	Roll Back Malaria
**SEG**	Socio-economic group
**SP**	Sulphadoxine/ sulfalene-Pyrimethamine
**STI**	Sexually Transmitted Disease
**US$**	United States Dollars
**WHO**	World Health Organization
